# Supplementary material for: Constructing temporal networks with bursty activity patterns
Source: Nat Commun. 2023 Nov 11;14:7311. doi: 10.1038/s41467-023-42868-1 (PMC10640578; doi:10.1038/s41467-023-42868-1)
Supplement: Supplementary file 1 — Supplementary Information [file 41467_2023_42868_MOESM1_ESM.pdf]

Supplemantary Information for

Constructing temporal networks with bursty activity patterns

Anzhi Sheng, Qi Su, Aming Li, Long Wang, and Joshua B. Plotkin

This PDF file includes:

Supplementary Note

Supplementary Figures 1 to 14

Supplementary Tables 1 to 2

Supplementary Note

1 Renewal process

The activation process of a node/link can be naturally modeled as a point process. The inter-event time (IET) of two consecutive activities is a random variable ξ, and we assume the IETs are independent of each other and are identically distributed with a nonnegative distribution. Such a point process is called a renewal process.

We set ξ to be lattice with $d = 1$, that is, the activation can only occur at positive integral moments, which leads to $\sum_{n=1}^{\infty} \mathbb{P}(\xi = n) = 1$. Therefore, the renewal process of a node/link is a discrete-time stochastic process $\{\Xi_n\}_{n\geq 0}$, where $\Xi_n = 1$ if the node/link is active at time n, otherwise $\Xi_n = 0$.

2 Temporal network construction based on static networks

We consider the construction of temporal networks based on a static undirected network. We use the probabilistic graphical model to describe the activation process of individual nodes/links in a network system at each time step and generate a stochastic process to formalize the construction process.

2.1 Two-node systems

2.1.1 Theoretical analysis

The elementary component of a network system is a two-node system, where two nodes x and y are connected by a link z (see Fig. 1a in the main text). The renewal processes of nodes x, y and link z are denoted as $\{X_n\}_{n\geq 0}$, $\{Y_n\}_{n\geq 0}$ and $\{Z_n\}_{n\geq 0}$. The initial states of the nodes and the link are all active (i.e. $X_0 = Y_0 = Z_0 = 1$), and for simplicity, we omit the specification of the initial state in the rest of this Supplementary Material unless otherwise specified.

The targeted IET distribution of node x is a probability mass function $F(n)$,

$$F(n) = \mathbb{P}(X_0 = 1, X_1 = 0, X_2 = 0, ..., X_n = 1), \quad n \in \mathbb{Z}^+, \tag{S1}$$

which represents the probability of the time interval between two consecutive activations. The probability mass function of node y and link z is denoted as $G(\Delta t)$ and $H(\Delta t)$, respectively. The trajectory of node x (y or z) until time n is denoted as $\mathbf{w}_x^{(n)} = (w_x^{(0)}, ..., w_x^{(n)})^{\mathrm{T}}$ ($\mathbf{w}_y^{(n)} =$

$(w_x^{(0)}, ..., w_x^{(n)})^{\mathrm{T}}$ or $\mathbf{w}_z^{(n)} = (w_z^{(0)}, ..., w_z^{(n)})^{\mathrm{T}}$), recording all $n+1$ historical states. We assume that link z is active at time n if and only if node x and y are all active at time n, which leads to

$$\mathbf{w}_z^{(n)} = \mathbf{w}_x^{(n)} \circ \mathbf{w}_y^{(n)},$$

where the operation $\circ$ is the Hadamard product.

Given the trajectory $\mathbf{w}_x^{(n-1)}$, the conditional probability that node x is active at time n is denoted as $p_x(\mathbf{w}_x^{(n-1)}, 1)$. To calculate the probability $p_x(\mathbf{w}_x^{(n-1)}, 1)$, we provide two identities by the property of renewal processes,

$$\begin{aligned}
&\mathbb{P}(X_n = 1 | X_{n-1} = 0, ..., X_1 = 0, X_0 = 1) = \frac{\mathbb{P}(X_0 = 1, X_1 = 0, X_2 = 0, ..., X_n = 1)}{\mathbb{P}(X_0 = 1, X_1 = 0, ...X_{n-1} = 0)} = \frac{F(n)}{\sum_{i \geq n} F(i)}, \\
&\mathbb{P}(X_n = a | X_m = 1, X_{m-1} = w_{m-1}, ..., X_1 = w_1, X_0 = 1) = \mathbb{P}(X_{n-m} = a | X_0 = 1), \quad a \in \{0, 1\}.
\end{aligned}$$

The first equation shows the value of the conditional probability that x is active at time n, given that x is inactive at all previous moments (except the initial moment), and the second equation illustrates the state of x at the current moment is determined by all historical states from the last activation moment.

We denote the random vector $(X_0, X_1, ..., X_n)^{\mathrm{T}}$ by $\mathbf{X}^{(n)}$, the conditional probability $p_x(\mathbf{w}_x^{(n-1)}, a)$ is given by

$$p_x(\mathbf{w}_x^{(n-1)}, a) = \mathbb{P}(X_n = 1 | \mathbf{X}^{(n-1)} = \mathbf{w}_x^{(n-1)}) = \begin{cases} \dfrac{F(n-m)}{\sum_{i \geq n-m} F(i)}, & a = 1, \\ \dfrac{\sum_{i \geq n-m+1} F(i)}{\sum_{i \geq n-m} F(i)}, & a = 0, \end{cases} \tag{S2}$$

where $m = \max\{k \leq n : w_x^{(k)} = 1\}$ is the last activation moment of x. The same conclusions can be obtained for node y and link z.

The construction algorithm of a two-node system is formalized by a stochastic process $\{S_n\}_{n \geq 0}$, satisfying for arbitrary sets of $t_1, ..., t_k \in \mathbb{N}, k \in \mathbb{Z}^+$,

$$\begin{aligned}
&\mu_{S_{2t_1}, ..., S_{2t_k}} = \mu_{X_{t_1}, ..., X_{t_k}}, \\
&\mu_{S_{2t_1+1}, ..., S_{2t_k+1}} = \mu_{Y_{t_1}, ..., Y_{t_k}}, \\
&\mu_{(S_{2t_1} \cdot S_{2t_1+1}), ..., (S_{2t_k} \cdot S_{2t_k+1})} = \mu_{Z_{t_1}, ..., Z_{t_k}},
\end{aligned} \tag{S3}$$

and $S_0 = S_1 = 1$, where $\mu_{X_{t_1}, ..., X_{t_k}}$ represents the finite dimensional distribution of $\{S_n\}_{n \geq 0}$ at the time slice $(t_1, ..., t_k)$.

To prove the existence of $\{S_n\}_{n \geq 0}$, we need to specify all finite dimensional distributions of $\{S_n\}_{n \geq 0}$. We assume X_{n+1} and $\mathbf{Y}^{(n)}$ be conditionally independent with respect to $\mathbf{X}^{(n)}$, that

is,

$$\mathbb{P}(X_{n+1}, \mathbf{Y}^{(n)} | \mathbf{X}^{(n)}) = \mathbb{P}(X_{n+1} | \mathbf{X}^{(n)}) \cdot \mathbb{P}(\mathbf{Y}^{(n)} | \mathbf{X}^{(n)}), \tag{S4}$$

which means that the state of x at the current moment is unrelated to the past of y. By symmetry, we have

$$\mathbb{P}(Y_{n+1}, \mathbf{X}^{(n)} | \mathbf{Y}^{(n)}) = \mathbb{P}(Y_{n+1} | \mathbf{Y}^{(n)}) \cdot \mathbb{P}(\mathbf{X}^{(n)} | \mathbf{Y}^{(n)}).$$

When $X_{n+1} = a$ and $\mathbf{X}^{(n)} = \mathbf{w}_x^{(n)}$, using Eqs. (S3) and (S4),

$$\begin{aligned} \mathbb{P}(S_{2n+2} = a | S^{(2n+1)}) = \mathbb{P}(X_{n+1} = a | \mathbf{X}^{(n)}, \mathbf{Y}^{(n)}) &= \frac{\mathbb{P}(X_{n+1} = a, \mathbf{Y}^{(n)} | \mathbf{X}^{(n)}) \cdot \mathbb{P}(X^n)}{\mathbb{P}(\mathbf{Y}^{(n)} | \mathbf{X}^{(n)}) \cdot \mathbb{P}(X^n)} \\ &= \frac{\mathbb{P}(X_{n+1} = a | \mathbf{X}^{(n)}) \cdot \mathbb{P}(\mathbf{Y}^{(n)} | \mathbf{X}^{(n)})}{\mathbb{P}(\mathbf{Y}^{(n)} | \mathbf{X}^{(n)})} = \mathbb{P}(X_{n+1} = a | \mathbf{X}^{(n)}) \\ &= p_x(\mathbf{w}_x^{(n)}, a), \end{aligned} \tag{S5}$$

and

$$\begin{aligned} \mathbb{P}(S_{2n+3} = b | S^{(2n+2)}) = \mathbb{P}(Y_{n+1} = b | X^{(n+1)}, \mathbf{Y}^{(n)}) &= \frac{\mathbb{P}(X^{(n+1)}, Y^{(n+1)})}{\mathbb{P}(X^{(n+1)}, \mathbf{Y}^{(n)})} \\ &= \frac{\mathbb{P}(X_{n+1} = a, Y_{n+1} = b | \mathbf{X}^{(n)}, \mathbf{Y}^{(n)}) \mathbb{P}(\mathbf{X}^{(n)}, \mathbf{Y}^{(n)})}{\mathbb{P}(X_{n+1} = a | \mathbf{X}^{(n)}, \mathbf{Y}^{(n)}) \mathbb{P}(\mathbf{X}^{(n)}, \mathbf{Y}^{(n)})} \\ &= \frac{\mathbb{P}(X_{n+1} = a, Y_{n+1} = b | \mathbf{X}^{(n)}, \mathbf{Y}^{(n)})}{p_x(\mathbf{w}_x^{(n)}, a)}. \end{aligned} \tag{S6}$$

The numerator $\mathbb{P}(X_{n+1}, Y_{n+1} | \mathbf{X}^{(n)}, \mathbf{Y}^{(n)})$ in Eq. (S6) is related to $\mathbf{w}_x^{(n)}$, $\mathbf{w}_y^{(n)}$ and $\mathbf{w}_z^{(n)}$, which can be calculated as

$$\mathbb{P}(X_{n+1} = 1, Y_{n+1} = 1 | \mathbf{X}^{(n)}, \mathbf{Y}^{(n)}) = p_z(\mathbf{w}_z^{(n)}, 1), \tag{S7a}$$

$$\mathbb{P}(X_{n+1} = 1, Y_{n+1} = 0 | \mathbf{X}^{(n)}, \mathbf{Y}^{(n)}) = p_x(\mathbf{w}_x^{(n)}, 1) - p_z(\mathbf{w}_z^{(n)}, 1), \tag{S7b}$$

$$\mathbb{P}(X_{n+1} = 0, Y_{n+1} = 1 | \mathbf{X}^{(n)}, \mathbf{Y}^{(n)}) = p_y(\mathbf{w}_y^{(n)}, 1) - p_z(\mathbf{w}_z^{(n)}, 1), \tag{S7c}$$

$$\mathbb{P}(X_{n+1} = 0, Y_{n+1} = 0 | \mathbf{X}^{(n)}, \mathbf{Y}^{(n)}) = 1 + p_z(\mathbf{w}_z^{(n)}, 1) - p_x(\mathbf{w}_x^{(n)}, 1) - p_y(\mathbf{w}_y^{(n)}, 1). \tag{S7d}$$

When $\mathbb{P}(B) = 0$, we impose the conditional probability $\mathbb{P}(A|B) = 0$. Using Eqs. (S3)-(S7), we can calculate all finite dimensional distributions of $\{S_n\}_{n \geq 0}$ and verify that they satisfy the suitable consistency conditions. By the Kolmogorov extension theorem [1], a probability space $(\Omega, \mathcal{F}, \mathbb{P})$ exists such that $\{S_n\}_{n \geq 0}$ is well-defined on this probability space.

When $F = G = H$, we obtain that Eqs. (S7b) and (S7c) are equal to 0, which indicates that the state of x, y, and z is the same at any time step. In this case, we say that the system is synchronous.

2.1.2 Consistency condition

Mathematically, some distribution combinations F, G, H cannot lead to a well-defined stochastic process $\{S_n\}_{n\geq 0}$. The reason is that the values of Eqs. (S7a)-(S7d) might be less than 0 and therefore violate the definition of probability measure. Here, we propose a definition of distribution consistency F, G, H.

Definition 1 (Distribution consistency)**.** *The distributions $F(n)$, $G(n)$ and $H(n)$ are said to be consistent if the values Eqs. (S7a)-(S7d) all belong to $[0,1]$ for any possible $\boldsymbol{w}_x^{(n)}, \boldsymbol{w}_y^{(n)}$, $n \geq 0$.*

We also define the consistency of a two-node system.

Definition 2 (Two-node system consistency)**.** *The two-node system is said to be consistent if the targeted distribution of the two nodes and the link are consistent.*

When a system is consistent, the IET distribution of each node (link) fulfills the targeted distribution. Here, we first propose a necessary condition to verify system consistency with general distributions and analyze three classes of distributions – power-law distributions, (discrete) exponential distributions, and Poisson distributions. All conclusions are based on the premise that the length of temporal networks t_{tol} is free.

The necessary condition is that the support set for H needs to be a denumerable set, i.e., $\forall m \geq 1$, $\exists n \geq m$, $H(n) > 0$. If not, there exists a positive integer m, such that $H(m) > 0$ and $H(n) = 0$, for all $n > m$. When

$$\mathbf{w}_z^{(m-1)} = \mathbf{0}^{(m-1)} = (1, \underbrace{0, ..., 0}_{m-1})^{\mathrm{T}}, \tag{S8}$$

the identity $p_z(\mathbf{w}_z^{(m-1)}, 1) = 1$ holds, meaning that link z is almost surely active at time m. However, it is impossible to find F and G such that $p_x(\mathbf{w}_x^{(m-1)}, 1) = p_y(\mathbf{w}_y^{(m-1)}, 1) = 1$ holds for all $\mathbf{w}_x^{(m-1)} \circ \mathbf{w}_y^{(m-1)} = \mathbf{0}^{(m-1)}$. In other words, the probability $p_x(\mathbf{w}_x^{(m-1)}, 1)$ or $p_y(\mathbf{w}_y^{(m-1)}, 1)$ would be smaller than $p_z(\mathbf{w}_z^{(m-1)}, 1)$ under some trajectories, which results in Eq. (S7b) or (S7c) being smaller than 0.

Next, we consider that F, G and H are all power-law distributions, that is,

$$F(n) = C_1 n^{-\alpha_1},\ G(n) = C_2 n^{-\alpha_2},\ H(n) = C_3 n^{-\beta},$$

where α_1, α_2, β are the exponents larger than 1, and $C_i = (\sum_{n=1}^{\infty} n^{-\alpha_i})^{-1}$ $(i = 1, 2, 3)$ are the normalization constants. The conditional probability of x being active for the first time at

time n is given by

$$
\begin{aligned}
p_x^{(n)} = p_x(\mathbf{0}^{(n-1)}, 1) &= \frac{F(n)}{\sum_{i\geq n} F(i)} \approx \frac{C_1 \int_{n-1/2}^{n+1/2} x^{-\alpha_1} dx}{C_1 \int_{n-1/2}^{+\infty} x^{-\alpha_1} dx} \\
&= \frac{(n-\frac{1}{2})^{-\alpha_1+1} - (n+\frac{1}{2})^{-\alpha_1+1}}{(n-\frac{1}{2})^{-\alpha_1+1}} = 1 - (\frac{n+\frac{1}{2}}{n-\frac{1}{2}})^{-\alpha_1+1}.
\end{aligned}
\tag{S9}
$$

Equation (S9) shows that $p_x^{(n)}$ is monotonically decreasing/increasing with respect to n/α_1, and when $n \to \infty$, $p_x^{(n)} \to 0$. Since $w_x^{(i)} \geq w_z^{(i)}$ and $w_y^{(i)} \geq w_z^{(i)}$ for all $i \geq 0$, by Eqs. (S7b) and (S7c), the exponent of the nodes need to be not less than that of the link, $\alpha_1 \geq \beta$ and $\alpha_2 \geq \beta$. In addition, a necessary and sufficient condition for Eq. (S7d) belonging to $[0, 1]$ for all trajectories is

$$
p_x^{(1)} + p_y^{(2)} < 1, \quad p_x^{(1)} + p_y^{(1)} < 1 + p_z^{(1)}.
$$

In particular, when all nodes have the same exponent α, the first condition becomes $p_x^{(1)} + p_x^{(2)} < 1$ and the second condition becomes $2C_1 < 1 + C_3$.

The exponential distribution we discuss is a discrete exponential distribution, and the analytical forms of F, H, G are given by

$$
F(n) = C_1 \int_{n-1/2}^{n+1/2} \lambda_1 e^{-\lambda_1 x} \mathrm{d}x, \; G(n) = C_2 \int_{n-1/2}^{n+1/2} \lambda_2 e^{-\lambda_2 x} \mathrm{d}x, \; H(n) = C_3 \int_{n-1/2}^{n+1/2} \lambda_3 e^{-\lambda_3 x} \mathrm{d}x,
$$

where λ_i ($i = 1, 2, 3$) are the exponents and $C_i = e^{\frac{\lambda_i}{2}}$ ($i = 1, 2, 3$) are the normalization constants.

$$
p_x^{(n)} = \frac{C_1 \int_{n-1/2}^{n+1/2} \lambda_1 e^{-\lambda_1 x} \mathrm{d}x}{C_1 \int_{n-1/2}^{+\infty} \lambda_1 e^{-\lambda_1 x} \mathrm{d}x} = \frac{e^{-\lambda_1 (n-\frac{1}{2})} - e^{-\lambda_1 (n+\frac{1}{2})}}{e^{-\lambda_1 (n-\frac{1}{2})}} = 1 - e^{-\lambda_1}. \tag{S10}
$$

Equation (S10) shows that $p_x^{(n)}$ is monotonically increasing with respect to λ_1 and independent to n. To satisfy the non-negativity of Eqs. (S7a) and (S7b), the exponent of the nodes need to be not less than the link (i.e. $\lambda_1 \geq \lambda_3$, $\lambda_2 \geq \lambda_3$). To satisfy the non-negativity of Eq. (S7d), the necessary and sufficient condition is $e^{-\lambda_1} + e^{-\lambda_2} \geq e^{-\lambda_3}$. When two nodes have the same exponent, the conditions simplifies to $0 \leq \lambda_{node} - \lambda_{link} \leq \ln 2$, which means nodes need to be more frequently active than links (the lower bound), while not too frequently (the upper bound).

Another common discrete distribution defined over $\mathbb{N}$ is the Poisson distribution. We prove that when F, G, and H are all Poisson distributions, the system is inconsistent. Since $n \geq 1$, we modify the definition domain of the Poisson distribution, $\mathbb{P}(X = n) = e^{-\lambda} \frac{\lambda^{n-1}}{(n-1)!}$, $\forall n \in \mathbb{Z}^+$. We have

$$
p_x^{(n)} = \frac{e^{-\lambda} \frac{\lambda^{n-1}}{(n-1)!}}{\sum_{i\geq n} e^{-\lambda} \frac{\lambda^{i-1}}{(i-1)!}} = \frac{1}{1 + \sum_{i\geq 1} \frac{\lambda^i}{(n+i)!/n!}} = \frac{1}{1 + \frac{\lambda}{n} + \frac{\lambda^2}{(n+1)n} + \ldots}. \tag{S11}
$$

Algorithm 1 Construction on two-node systems

Input: IET distributions $F(\Delta t)$, $G(\Delta t)$, $H(\Delta t)$ and parameter t_{tol}
Output: trajectories $\mathbf{w}_x^{t_{tol}}$, $\mathbf{w}_y^{t_{tol}}$, $\mathbf{w}_z^{t_{tol}}$ or 0

for $t = 1$ to t_{tol} **do**
  Compute $p(\mathbf{w}_x^{(t-1)}, 1)$, $p(\mathbf{w}_y^{(t-1)}, 1)$ and $p(\mathbf{w}_z^{(t-1)}, 1)$
  Compute $p_1 = p_z(\mathbf{w}_z^{(t-1)}, 1)$, $p_2 = p_x(\mathbf{w}_x^{(t-1)}, 1) - p_z(\mathbf{w}_z^{(t-1)}, 1)$, $p_3 = p_y(\mathbf{w}_y^{(t-1)}, 1) - p_z(\mathbf{w}_z^{(t-1)}, 1)$, and $p_4 = 1 + p_z(\mathbf{w}_z^{(t-1)}, 1) - p_x(\mathbf{w}_x^{(t-1)}, 1) - p_y(\mathbf{w}_y^{(t-1)}, 1)$
  if $p_1 < 0$ or $p_2 < 0$ or $p_3 < 0$ or $p_4 < 0$ **then**
    return 0
  Choose two independent random numbers p, q uniformly in $[0, 1]$
  if $p < p(\mathbf{w}_x^{(t-1)}, 1)$ **then**
    $w_x^{(t)} = 1$
    $r = p_1 / p(\mathbf{w}_x^{(t-1)}, 1)$
    if $q < r$ **then**
      $w_y^{(t)} = 1$
    else
      $w_y^{(t)} = 0$
  else
    $w_x^{(t)} = 0$
    $r = p_3 / (1 - p(\mathbf{w}_x^{(t-1)}, 1))$
    if $q < r$ **then**
      $w_y^{(t)} = 1$
    else
      $w_y^{(t)} = 0$
  $w_z^{(t)} = w_x^{(t)} \cdot w_y^{(t)}$
return $\mathbf{w}_x^{t_{tol}}$, $\mathbf{w}_y^{t_{tol}}$, $\mathbf{w}_z^{t_{tol}}$

Equation (S11) shows that $p_x^{(n)}$ is monotonically decreasing with respect to λ and monotonically increasing with respect to n, when $n \to \infty$, $p_x^{(n)} \to 1$. Therefore, for all $\lambda_{node}, \lambda_{link}$, a positive integer K exists such that $p_H^{(K)} > p_F^{(1)}$, which indicates that the value of Eq. (S7b) is less than 0.

For a more general combination (F, G, H), we need to calculate the result of Eq. (S7) at each time step, and once one of the probabilities in Eq. (S7) is less than 0, the construction stops, and the system is inconsistent.

2.1.3 Construction algorithm

Algorithm 1 describes the procedure of two-node temporal network construction. At each time step, we first determine the activation of x which is only related to the trajectory of x. Then we decide the state of y. Finally, the state of z is determined by the states of x and y. If the return of the algorithm is 0, then the system is inconsistent. Otherwise, the IET

distributions of x, y and z fulfill F, G and H.

2.2 Tree systems

A natural extension of two-node systems is tree systems (see Fig. 1b in the main text). Here we give a theoretical explanation and an explicit algorithm for the construction of tree structures.

2.2.1 Theoretical analysis

A tree system with N nodes is denoted as $\mathcal{G} = (\mathcal{V}, \mathcal{E})$, where $\mathcal{V} = \{0, ..., N-1\}$ is a set of nodes and $\mathcal{E} = \{(i,j) : i,j \in \mathcal{V}\}$ is a set of links. The renewal process of node i and link (m,n) is denoted as $\{O_n^{(i)}\}_{n\geq 0}$ and $\{E_n^{(m,n)}\}_{n\geq 0}$, respectively. We randomly select a node $r \in \mathcal{V}$ as the root of the tree and classify all other nodes according to their distance from r. All nodes with l steps away from r compose the lth layer of the tree. The number of layers is denoted as l_{tol}, and the number of nodes in each layer l is denoted as n_l.

We define a mapping v from the original set of nodes $\mathcal{V}$ to the new one,

$$v : j \to (l, m),$$

where $j \in \mathcal{V}$ is the original serial number, l is the distance from j to $\mathbf{r}$ and m is the number of j in the layer l. For example, we have $v(r) = (0, 1)$. We define a partial ordering $\preccurlyeq$ on $v(\mathcal{V})$, the relation $(i_1, j_1) \preccurlyeq (i_2, j_2)$ holds when $i_1 < i_2$ or $i_1 = i_2, j_1 \leq j_2$. Based on the relationship $\preccurlyeq$, we order the elements of $v(\mathcal{V})$. We use $\tilde{v}$ to represent this sorting,

$$\tilde{v} : v(\mathcal{V}) \to \mathcal{V}.$$

The composite mapping $\hat{v} = \tilde{v} \circ v : \mathcal{V} \to \mathcal{V}$ represents the reordering of $\mathcal{V}$, and the result is denoted as $\widetilde{\mathcal{V}}$.

For the link set $\mathcal{E}$, we also define a reordering mapping $\hat{e}$,

$$\hat{e} : (x, y) \to (p, q),$$

where $p = \hat{v}(x)$, $q = \hat{v}(y)$.

We set $u = \hat{u}^{-1}$ and $f = \hat{e}^{-1}$. Similar to the two-node system, we construct a new stochastic

process $\{T_n\}_{n\geq 0}$ that for arbitrary sets of $t_1, ..., t_k \in \mathbb{N}$, $k \in \mathbb{Z}^+$ satisfies

$$\mu_{T_{Nt_1+i},...,T_{Nt_k+i}} = \mu_{O_{t_1}^{u(i)},...,O_{t_k}^{u(i)}}, \quad \forall i \in \{0, ..., N-1\}, \tag{S12a}$$

$$\mu_{(T_{Nt_1+i}\cdot T_{Nt_1+j}),...,(T_{Nt_k+i}\cdot T_{Nt_k+j})} = \mu_{E_{t_1}^{f(i,j)},...,E_{t_k}^{f(i,j)}}, \quad \forall (i,j) \in \hat{e}(\mathcal{E}), \tag{S12b}$$

$$T_k = 1, \ \forall k \in \{0, ..., N-1\}, \tag{S12c}$$

where $\mu_{O_{t_1}^{u(i)},...,O_{t_k}^{u(i)}}$ and $\mu_{E_{t_1}^{f(i,j)},...,E_{t_k}^{f(i,j)}}$ are the finite dimensional distributions about node $u(i) \in \mathcal{V}$ and link $f(i,j) \in \mathcal{E}$ at the time slice $(t_1, ..., t_k)$, respectively.

Equations (S12a)-(S12c) illustrate that the marginal distributions of $\{T_n\}_{n\geq 0}$ satisfy the corresponding distributions of single nodes or single links. We can simply consider that $\{T_n\}_{n\geq 0}$ consists of the following sequence

$$(T_0, ..., T_{N-1}, ..., T_{Nk}, ..., T_{N(k+1)-1}, ...) = (O_0^{u(0)}, ..., O_0^{u(N-1)}, ..., O_k^{u(0)}, ..., O_k^{u(N-1)}, ...),$$

and $T_{Nk+i} \cdot T_{Nk+j} = E_k^{f(i,j)}$.

We continue with the previous notation $T^{(Nk-1)} = (T_0, ..., T_{Nk-1})$. To prove the existence of $\{T_n\}_{n\geq 0}$, we specify all finite dimensional distributions of $\{T_n\}_{n\geq 0}$. We assume the following conditional independence, for all $k \in \mathbb{N}$,

$$\mathbb{P}(T_{Nk+i}|T^{(Nk-1)}) = \mathbb{P}(O_k^{u(i)}|O_{k-1}^{u(i)}, ..., O_0^{u(i)}), \tag{S13}$$

and for arbitrary mutually unequal $i_1, ..., i_n, i, m \in \mathbb{N}$ that satisfy: (1) $(i_c, i) \notin \hat{e}(\mathcal{E})$, $\forall c \in \{1, ..., k\}$, (2) $(i, m) \in \hat{e}(\mathcal{E})$, we have

$$\mathbb{P}(T_{Nk+i}|T_{Nk+m}, T_{Nk+i_1}, ..., T_{Nk+i_n}, T^{(Nk-1)}) = \mathbb{P}(O_k^{u(i)}|O_{k-1}^{u(i)}, ..., O_0^{u(i)}, O_k^{u(m)}, ..., O_0^{u(m)}). \tag{S14}$$

Equation (S13) illustrates that, given the historical trajectory of the other nodes, the activation of node i at the current state is only related to its own trajectory. Equation (S14) indicates that the activation of node i only depends on the two-node system in which it is located, given the trajectory of another node in the same two-node system.

A corollary of Eq. (S14) is that for arbitrary mutually unequal $i_1, ..., i_n, i, j, m \in \mathbb{N}$ that satisfy: (1) $(i_c, i), (i_c, j) \notin \hat{e}(\mathcal{E})$, $\forall c \in \{1, ..., k\}$, (2) $(i, m), (j, m) \in \hat{e}(\mathcal{E})$,

$$\begin{aligned}\mathbb{P}(T_{Nk+i}, T_{Nk+j}|T_{Nk+m}, T_{Nk+i_1}, ..., T_{Nk+i_n}, T^{(Nk-1)}) = {} & \mathbb{P}(O_k^{u(i)}|O_{k-1}^{u(i)}, ..., O_0^{u(i)}, O_k^{u(m)}, ..., O_0^{u(m)}) \\ & \times \mathbb{P}(O_k^{u(j)}|O_{k-1}^{u(j)}, ..., O_0^{u(j)}, O_k^{u(m)}, ..., O_0^{u(m)}).\end{aligned} \tag{S15}$$

Equation (S15) demonstrates the conditional independence for the triplets (r,a1,b1) and (r,a1,a2) shown in Fig. 1b in the main text.

Algorithm 2 Construction on tree systems

Input: tree structure $\mathcal{T}$, IET distributions for all nodes and links, and parameter t_{tol}
Output: trajectories of all nodes and links

for $t = 1$ to t_{tol} **do**
  Select the root r and label all other nodes as (l, m) according to the distance from r
  $p_{\mathbf{r}} = p(\mathbf{w}_{(0,1)}^{(t-1)}, 1)$ $(l = 0)$
  Select a random number q uniformly in $[0, 1]$
  if $q < p_{\mathbf{r}}$ **then**
    $w_{\mathbf{r}}^{(t)} = 1$
  else
    $w_{\mathbf{r}}^{(t)} = 0$
  for $l = 1$ to l_{tol} **do**
    for $m = 1$ to n_l **do**
      Search k, such that $x = v^{-1}(l-1, k)$, $y = v^{-1}(l, m)$, $z = (x, y) \in \mathcal{E}$
      Execute a single loop of Algorithm 1 on the two-node system consisting of
      the nodes x, y and the link z
return trajectories of all nodes and links

Using Eqs. (S12)-(S14), we can calculate all finite dimensional distributions of $\{T_n\}_{n\geq 0}$, then the existence of $\{T_n\}_{n\geq 0}$ can be proved with the Kolmogorov extension theorem [1].

2.2.2 Consistency condition

We propose the definition of the consistency of a tree system.

Definition 3 (Tree system consistency)**.** *A tree system is said to be consistent if all two-node systems in the tree system are consistent.*

This definition shows that the consistency of a tree system is determined by the consistency of each two-node system.

Similar to two-node systems, we also have equivalents for the system consistency under different activity patterns. When the targeted distributions are all power-law distributions, the necessary and sufficient condition is that each two-node system satisfies $p_x^{(1)} + p_y^{(2)} < 1$, $p_x^{(1)} + p_y^{(1)} < 1 + p_z^{(1)}$, and the exponents of nodes are larger than the link. When the targeted distributions are discrete exponential distributions, the necessary and sufficient condition is that the difference between the exponents of each node and each link is between 0 and $\ln 2$ in each two-node system. When the targeted distributions are Poisson distributions, the tree system is inconsistent.

2.2.3 Construction algorithm

Algorithm 2 shows the construction of temporal networks on tree systems. At each time step, the state of the root r is updated according to its own trajectory. Then the state of leaves is updated sequentially by executing Algorithm 1 on every two-node system. For each loop in lines 10-13, the state of all nodes on layer l is updated. Specifically, to determine the state of y on layer l, we first identify the node x on layer $l-1$, which is directly connected to y. At this point, the state of x has already been updated. Then, the state of y is updated within the two-node system formed by x and y.

2.3 Arbitrary structured systems

We can always find a spanning tree for any network structure. We call a link a *trunk* if it is in the spanning tree, a *branch* otherwise. The activity of a network system is determined by its spanning tree. Algorithm 3 shows the construction of temporal networks on arbitrary underlying topologies $\mathcal{G}$. In the algorithm, we first select a spanning tree of $\mathcal{G}$. At each time step, we execute Algorithm 2 on the spanning tree, so that the states of all nodes and trunks are updated. Then, the state of each branch is determined according to the state of the nodes on both ends.

Since the activity of a system is established by the activity of its spanning tree system, we give the following definition,

Definition 4 (Arbitrary system consistency)**.** *The two-node system is said to be consistent if the targeted distribution of the two nodes and the link are consistent.*

In particular, when all nodes and trunks have the same targeted IET distribution, the second and third probabilities in Eq. (S7) are always equal to 0 for any two-node system, which means that the nodes and trunks are active or inactive at the same time. As a result, the whole system is synchronous, i.e. all nodes and links (including branches) always become active or inactive at the same time. This property ensures that, for any targeted distributions of nodes and links (fulfilling the consistency condition), we can always find inputs for nodes and trunks, such that the algorithmic IET distributions match with the target ones.

2.4 Systems with ring structure

A significant property of a tree system $\mathcal{T} = (\mathcal{V}, \mathcal{E})$ is that there is no ring structure, that is, for all $i_1, ..., i_m \in \mathcal{V}$, $a_{i_1 i_2} \times ... \times a_{i_{m-1} i_m} \times a_{i_m i_1} = 0$, where $a_{ij} = 1$ if $(i, j) \in \mathcal{E}$, otherwise $a_{ij} = 0$. For a general network structure, the ring structure exists and one of the links in the ring would be a branch.

Algorithm 3 Construction based on arbitrary network systems

Input: network $\mathcal{G}$, IET distributions of all nodes and trunks, and parameter t_{tol}
Output: trajectories of all nodes and links

1: Select a spanning tree $\mathcal{T}$ of $\mathcal{G}$
2: Assign a probability mass function to each node and each link in $\mathcal{T}$
3: **for** $t = 1$ to t_{tol} **do**
4: Execute a single loop of Algorithm 2 for $\mathcal{T}$
5: Update the state of each branch according to the state of the nodes on both sides
return trajectories of all nodes and links

Below, we analyze the activity of branches. We consider the simplest system with a ring, which consists of three nodes, $\{x, y, z\}$, and three links, $\{(x, y), (y, z), (z, x)\}$. The renewal process of nodes x, y and z is denoted as $\{X_n\}_{n\geq 0}$, $\{Y_n\}_{n\geq 0}$ and $\{Z_n\}_{n\geq 0}$. We set x as the root r. At time $n+1$, the probability of x being active is $p(\mathbf{w}_x^{(n)}, 1)$ and the result is denoted as a. To make the IET distribution of node y and link (x, y) fulfill targeted distributions, the probability of y being active is

$$\frac{\mathbb{P}(X_{n+1} = a, Y_{n+1} = 1 | \mathbf{X}^{(n)} = \mathbf{w}_x^{(n)}, \mathbf{Y}^{(n)} = \mathbf{w}_y^{(n)})}{p(\mathbf{w}_x^{(n)}, a)}.$$

This result is denoted as b. Then, we can calculate the probability of z being active through the structure $\{y, (y, z), z\}$ or $\{x, (x, z), z\}$. The former leads to the probability

$$\frac{\mathbb{P}(Y_{n+1} = b, Z_{n+1} = 1 | \mathbf{Y}^{(n)} = \mathbf{w}_y^{(n)}, \mathbf{Z}^{(n)} = \mathbf{w}_z^{(n)})}{p(\mathbf{w}_y^{(n)}, b)}, \tag{S16}$$

and the latter leads to the probability

$$\frac{\mathbb{P}(X_{n+1} = a, Z_{n+1} = 1 | \mathbf{X}^{(n)} = \mathbf{w}_x^{(n)}, \mathbf{Z}^{(n)} = \mathbf{w}_z^{(n)})}{p(\mathbf{w}_x^{(n)}, a)}. \tag{S17}$$

These two probabilities are not always the same. Using Eq. (S16) (Eq. (S17)) to obtain the state of z is equivalent to the select the spanning tree with links $(x, y), (y, z)$ ($(x, y), (x, z)$), and the state of the remaining link (i.e. the branch) is then established.

Furthermore, in general, the activity of branches is not a (strict) renewal process. Without loss of generality, we assume that links $(x, y), (x, z)$ are in the spanning tree. We denote the stochastic process of link (y, z) as $\{S_n\}_{n\geq 0}$. To prove the above assertion, we verify a sufficient condition

$$\mathbb{P}(S_2 = 1 | S_1 = 1) \neq \mathbb{P}(S_1 = 1). \tag{S18}$$

The left-hand side of Eq. (S18) equals

$$\frac{1}{\mathbb{P}(S_1=1)}\sum_{x_1,x_2\in\{0,1\}}\mathbb{P}(X_1=x_1,Y_1=1,Z_1=1,X_2=x_2,Y_2=1,Z_2=1). \tag{S19}$$

The numerator of Eq. (S19) equals

$$\sum_{x_1\in\{0,1\}}\mathbb{P}(X_1=x_1,Y_1=1,Z_1=1)\sum_{x_2\in\{0,1\}}\begin{pmatrix}\mathbb{P}(X_2=x_2,Y_2=1|X_1=x_1,Y_1=1)\\ \times\mathbb{P}(X_2=x_2,Z_2=1|X_1=x_1,Z_1=1)\end{pmatrix}/\mathbb{P}(X_2=x_2|X_1=x_1). \tag{S20}$$

In general, Eq. (S20) is not equal to

$$\sum_{x_1\in\{0,1\}}\mathbb{P}(X_1=x_1,Y_1=1,Z_1=1)\sum_{x_2\in\{0,1\}}\mathbb{P}(X_1=x_2,Y_1=1,Z_1=1)=\mathbb{P}(S_1=1)^2,$$

which means that Eq. (S18) holds. However, we can still use renewal processes to approximate the activity of branches (see fig. S6 and section 4 for reasons)

3 Robustness analysis

In the main text, we find that the aggregated IET distributions of nodes and links are robust to underlying topologies and the selection of spanning trees. The main reason is that the IET distribution of branches is only sensitive to targeted distributions. Specifically, we prove that the algorithmic distribution of every single branch is an exponential distribution (a heavy-tailed distribution) in Poisson-like activity patterns (bursty activity patterns) with upper and lower bounds determined by algorithm inputs. Here, we provide theoretical explanations.

3.1 Activity of branches in Poisson-like activity patterns

We begin our analysis with a three-node ring consisting of three nodes x, y, z and three links (x,y),(y,z),(z,x). We select a spanning tree with links (x,y) and (y,z), and the root is node x. The renewal process of nodes x,y,z is denoted as $\{X_n\}_{n\geq 0}$, $\{Y_n\}_{n\geq 0}$,$\{Z_n\}_{n\geq 0}$, and the exponent of nodes x,y,z (links $(x,y),(x,z)$) is $\lambda_x,\lambda_y,\lambda_z$ ($\lambda_{xy},\lambda_{xz}$). For simplicity, we make the following assumption:

Assumption 1: $\lambda_x=\lambda_y=\lambda_z=\lambda_1>0$ and $\lambda_{xy}=\lambda_{xz}=\lambda_2>0$.

Considering the consistency condition for Poisson-like activity patterns, the relation $0\leq \lambda_1-\lambda_2\leq \ln 2$ holds. The stochastic process of link (x,z) is denoted as $\{S_n\}_{n\geq 0}$.

We define a random variable

$$\tau_x = \inf\{n \geq 1 : X_n = 1\},$$

which is a stopping time for node x, indicating the first activation time of x. Similarly, the stopping times for nodes y, z and link (x, z) are denoted as τ_y, τ_z and τ_s, respectively. We approximate the activity of (x, z) by a renewal process with the distribution of τ_s as its IET distribution.

We first prove the following theorem,

Theorem 1. *For a three-node ring satisfying Assumption 1,*

$$\mathbb{P}(\tau_s > n) = e^{-\mu n},$$

where $\mu > 0$.

If Theorem 1 holds, the algorithmic IET distribution of the branch is also exponential.

Proof. By the definition of τ_s, we have

$$\begin{aligned}
\mathbb{P}(\tau_s > n) &= \sum_{\mathbf{a}^{(n)} \circ \mathbf{c}^{(n)} = \mathbf{0}^{(n)}} \mathbb{P}(\mathbf{X}^{(n)} = \mathbf{a}^{(n)}, \mathbf{Z}^{(n)} = \mathbf{c}^{(n)}) \\
&= \sum_{\mathbf{a}^{(n)} \circ \mathbf{c}^{(n)} = \mathbf{0}^{(n)}} \sum_{\mathbf{b}^{(n)} \in \{0,1\}^{n+1}} \mathbb{P}(\mathbf{X}^{(n)} = \mathbf{a}^{(n)}, \mathbf{Y}^{(n)} = \mathbf{b}^{(n)}, \mathbf{Z}^{(n)} = \mathbf{c}^{(n)}),
\end{aligned}$$

where $\mathbf{a}^{(n)} = (a_0, ..., a_n)^{\mathrm{T}}$, $\mathbf{b}^{(n)} = (b_0, ..., b_n)^{\mathrm{T}}$, and $\mathbf{c}^{(n)} = (c_0, ..., c_n)^{\mathrm{T}}$ are the trajectories of x, y, z with length $n + 1$, the definition of $\mathbf{0}^{(n)}$ is the same as that in Eq. (S8), and the operation $\circ$ is the Hadamard product.

For each fixed $\mathbf{a}^{(n)}, \mathbf{b}^{(n)}, \mathbf{c}^{(n)}$, using Eqs. (S13) and (S14), we have

$$\begin{aligned}
\mathbb{P}(\mathbf{X}^{(n)} = \mathbf{a}^{(n)}, \mathbf{Y}^{(n)} = \mathbf{b}^{(n)}, \mathbf{Z}^{(n)} = \mathbf{c}^{(n)}) = \mathbb{P}(X_n = a_n | \mathbf{X}^{(n-1)} = \mathbf{a}^{(n-1)}) \\
\times \mathbb{P}(Y_n = b_n | \mathbf{X}^{(n)} = \mathbf{a}^{(n)}, \mathbf{Y}^{(n-1)} = \mathbf{b}^{(n-1)}) \\
\times \mathbb{P}(Z_n = c_n | \mathbf{Y}^{(n)} = \mathbf{b}^{(n)}, \mathbf{Z}^{(n-1)} = \mathbf{c}^{(n-1)}) \\
\times \mathbb{P}(\mathbf{X}^{(n-1)} = \mathbf{a}^{(n-1)}, \mathbf{Y}^{(n-1)} = \mathbf{b}^{(n-1)}, \mathbf{Z}^{(n-1)} = \mathbf{c}^{(n-1)}).
\end{aligned} \tag{S21}$$

This gives

$$\mathbb{P}(\tau_s > n) = \sum_{\mathbf{a}^{(n-1)}\circ\mathbf{c}^{(n-1)}=\mathbf{0}^{(n-1)}} \sum_{\mathbf{b}^{(n-1)}\in\{0,1\}^n} \mathbb{P}(\mathbf{X}^{(n-1)} = \mathbf{a}^{(n-1)}, \mathbf{Y}^{(n-1)} = \mathbf{b}^{(n-1)}, \mathbf{Z}^{(n-1)} = \mathbf{c}^{(n-1)})$$
$$\times \sum_{a_n\times c_n=0} \sum_{b_n\in\{0,1\}} \Big(\mathbb{P}(X_n = a_n|\mathbf{X}^{(n-1)} = \mathbf{a}^{(n-1)})$$
$$\times \mathbb{P}(Y_n = b_n|\mathbf{X}^{(n)} = \mathbf{a}^{(n)}, \mathbf{Y}^{(n-1)} = \mathbf{b}^{(n-1)})$$
$$\times \mathbb{P}(Z_n = c_n|\mathbf{Y}^{(n)} = \mathbf{b}^{(n)}, \mathbf{Z}^{(n-1)} = \mathbf{c}^{(n-1)})\Big). \tag{S22}$$

Using Eqs. (S5)-(S7) and (S10), we have

$$\sum_{a_n\times c_n=0} \sum_{b_n\in\{0,1\}} \mathbb{P}(X_n = a_n|\mathbf{X}^{(n-1)} = \mathbf{a}^{(n-1)})$$
$$\times \mathbb{P}(Y_n = b_n|\mathbf{X}^{(n)} = \mathbf{a}^{(n)}, \mathbf{Y}^{(n-1)} = \mathbf{b}^{(n-1)})$$
$$\times \mathbb{P}(Z_n = c_n|\mathbf{X}^{(n)} = \mathbf{c}^{(n)}, \mathbf{Z}^{(n-1)} = \mathbf{b}^{(n-1)}) \tag{S23}$$
$$= e^{-\lambda_1} + \frac{(1-e^{-\lambda_2})(e^{-\lambda_2}-e^{-\lambda_1})}{1-e^{-\lambda_1}} + \frac{(e^{-\lambda_2}-e^{-\lambda_1})(2e^{-\lambda_1}-e^{-\lambda_2})}{e^{-\lambda_1}}$$
$$:= C(\lambda_1, \lambda_2).$$

This leads to

$$\mathbb{P}(\tau_s > n) = C\mathbb{P}(\tau_s > n-1).$$

Since $\mathbb{P}(\tau_s > 0)$ = 1, we have

$$\mathbb{P}(\tau_s > n) = C^n.$$

In this simplest ring, we can also analyze the decay of $\mathbb{P}(\tau_s > n)$ in three cases.

Case 1: When $\lambda_1 = \lambda_2$, we have $C = e^{-\lambda_1}$, which indicates that

$$\mathbb{P}(\tau_s > n) = e^{-\lambda_1 n} = \mathbb{P}(\mathbf{X}^{(n)} = \mathbf{0}^{(n)}, \mathbf{Y}^{(n)} = \mathbf{0}^{(n)}, \mathbf{Z}^{(n)} = \mathbf{0}^{(n)}).$$

This identity can also be obtained by the synchronization of the system because all elements have the same targeted distribution.

Case 2: When $\lambda_1 - \lambda_2 = \ln 2$, we have

$$C = \frac{e^{-\lambda_2}}{2} + \frac{(1-e^{-\lambda_2})e^{-\lambda_2}}{2-e^{-\lambda_2}} < 1,$$

meaning that the decay of $\mathbb{P}(\tau_s > n)$ is exponential.

Let $C = e^{-\mu}$, we compare the magnitude of $\mu(\lambda_2)$ and λ_2. Since

$$\frac{\mathrm{d}(\mu(\lambda_2) - \lambda_2)}{\mathrm{d}\lambda_2} = -\frac{2e^{\lambda_2}}{8e^{2\lambda_2} - 10e^{\lambda_2} + 3} < 0.$$

We get

$$\mu(\lambda_2) - \lambda_2 > 0, \quad \mu(\lambda_2) \to \lambda_2 \text{ as } \lambda_2 \to \infty.$$

This indicates that $\mathbb{P}(\tau_s > n)$ is also an exponential distribution, and the corresponding exponent is larger than that of single trunks, λ_2.

Case 3: When $0 < \lambda_1 - \lambda_2 < \ln 2$, let $x = e^{-\lambda_1}, y = e^{-\lambda_2}$, then

$$\partial_y C(x,y) = \frac{2(x^2 - 2x + y)}{x(x-1)}.$$

This gives

$$\max_y C(x,y) = (2-x)x < 1.$$

Similar to Case 2, let $C = e^{-\mu}$,

$$\frac{\mathrm{d}(\mu(\lambda_2) - \lambda_2)}{\mathrm{d}\lambda_2} = \frac{e^{3\lambda_1} + e^{2\lambda_2} - 2e^{\lambda_1+2\lambda_2}}{e^{3\lambda_1} - e^{2\lambda_2} + 2e^{\lambda_1+\lambda_2} - 4e^{2\lambda_1+\lambda_2} + 2e^{\lambda_1+2\lambda_2}}. \tag{S24}$$

For each fixed λ_1, the numerator and denominator of Eq. (S24) are quadratic functions with respect to e^{λ_2}. Considering $e^{\lambda_1}/2 < e^{\lambda_2} < e^{\lambda_1}$, when $\lambda_1 > \ln(3/2)$,

$$e^{3\lambda_1} - e^{2\lambda_2} + 2e^{\lambda_1+\lambda_2} - 4e^{2\lambda_1+\lambda_2} + 2e^{\lambda_1+2\lambda_2} < 0.$$

Then

$$\max_{\lambda_2}(\mu(\lambda_2) - \lambda_2) < \max\{\mu(\lambda_1) - \lambda_1, \mu(\lambda_1 - \ln 2) - \lambda_1 + \ln 2\} = 0.$$

This indicates that the exponent of $\mathbb{P}(\tau_s > n)$ is lower than λ_2.

□

For a general ring $\mathcal{R} = (\mathcal{V}, \mathcal{E})$, $\mathcal{V} = \{1, ..., m\}$ and $\mathcal{E} = \{(i,j) : i,j \in \mathcal{V}, |i-j| = 1\} \cup \{(1,m)\}$. The renewal process of individual nodes in $\mathcal{V}$ are denoted as $\{O_n^{(1)}\}_{n\geq 0}, \ldots, \{O_n^{(m)}\}_{n\geq 0}$. We add all links into the spanning tree except link $(1,m)$, and we denote the stochastic process of $(1,m)$ by $\{S_n\}_{n\geq 0}$. The first activation time of branch $(1,m)$ is also denoted as τ_s. We can prove the following theorem

Theorem 2. *For a general ring with m nodes, if every single node (trunk) has the same exponent, then*

$$\mathbb{P}(\tau_s > n) = e^{-\mu n},$$

where $\mu > 0$.

Proof. We denote the exponent of every single node (trunk) as λ_1 (λ_2). Let $\mathbf{O}^{(j,n)}$ and $\mathbf{i}^{(k,n)}$ represent $(O_0^{(j)}, ..., O_n^{(j)})$ and $(i_0^{(k)}, ..., i_n^{(k)})$, respectively. We have

$$\begin{aligned}\mathbb{P}(\tau_s > n) &= \sum_{\mathbf{i}^{(1,n)} \circ \mathbf{i}^{(m,n)} = \mathbf{0}^{(n)}} \mathbb{P}(\mathbf{O}^{(1,n)} = \mathbf{i}^{(1,n)}, \mathbf{O}^{(m,n)} = \mathbf{i}^{(m,n)}) \\ &= \sum_{\mathbf{i}^{(1,n)} \circ \mathbf{i}^{(m,n)} = \mathbf{0}^{(n)}} \sum_{\substack{\mathbf{i}^{(2,n)}, ..., \mathbf{i}^{(m-1,n)} \\ \in \{0,1\}^{n+1}}} \mathbb{P}(\mathbf{O}^{(1,n)} = \mathbf{i}^{(1,n)}, ..., \mathbf{O}^{(m,n)} = \mathbf{i}^{(m,n)}).\end{aligned}$$

For each fixed $\mathbf{i}^{(1,n)}, ..., \mathbf{i}^{(m,n)}$, similar to Eq. (S21), we have

$$\begin{aligned}\mathbb{P}(\mathbf{O}^{(1,n)} = \mathbf{i}^{(1,n)}, ..., \mathbf{O}^{(m,n)} = \mathbf{i}^{(m,n)}) = {} & \mathbb{P}(O_n^{(1)} = i_n^{(1)} | \mathbf{O}^{(1,n-1)} = \mathbf{i}^{(1,n-1)}) \\ & \times \mathbb{P}(O_n^{(2)} = i_n^{(2)} | \mathbf{O}^{(1,n)} = \mathbf{i}^{(1,n)}, \mathbf{O}^{(2,n-1)} = \mathbf{i}^{(2,n-1)}) \\ & \times ... \\ & \times \mathbb{P}(O_n^{(m)} = i_n^{(m)} | \mathbf{O}^{(m-1,n)} = \mathbf{i}^{(m-1,n)}, \mathbf{O}^{(m,n-1)} = \mathbf{i}^{(m,n-1)}) \\ & \times \mathbb{P}(\mathbf{O}^{(1,n-1)} = \mathbf{i}^{(1,n-1)}, ..., \mathbf{O}^{(m,n-1)} = \mathbf{i}^{(m,n-1)}),\end{aligned} \tag{S25}$$

meaning that node 1 is the root of the spanning tree. This gives

$$\begin{aligned}\mathbb{P}(\tau_s > n) = {} & \sum_{\substack{\mathbf{i}^{(1,n-1)} \\ \circ \mathbf{i}^{(m,n-1)} = \mathbf{0}^{(n-1)}}} \sum_{\substack{\mathbf{i}^{(2,n-1)}, ..., \mathbf{i}^{(m-1,n-1)} \\ \in \{0,1\}^n}} \mathbb{P}(\mathbf{O}^{(1,n-1)} = \mathbf{i}^{(1,n-1)}, ..., \mathbf{O}^{(m,n-1)} = \mathbf{i}^{(m,n-1)}) \\ & \times \sum_{i_n^{(1)} \times i_n^{(m)} = 0} \sum_{i_n^{(2)}, ..., i_n^{(m-1)} \in \{0,1\}} \mathbb{P}(O_n^{(1)} = i_n^{(1)} | \mathbf{O}^{(1,n-1)} = \mathbf{i}^{(1,n-1)}) \\ & \times \mathbb{P}(O_n^{(2)} = i_n^{(2)} | \mathbf{O}^{(1,n)} = \mathbf{i}^{(1,n)}, \mathbf{O}^{(2,n-1)} = \mathbf{i}^{(2,n-1)}) \\ & \times ... \\ & \times \mathbb{P}(O_n^{(m)} = i_n^{(m)} | \mathbf{O}^{(m-1,n)} = \mathbf{i}^{(m-1,n)}, \mathbf{O}^{(m,n-1)} = \mathbf{i}^{(m,n-1)}).\end{aligned} \tag{S26}$$

Using Eqs. (S5)-(S7) and (S10), we have

$$\begin{aligned}\sum_{i_n^{(1)} \times i_n^{(m)} = 0} \sum_{i_n^{(2)}, ..., i_n^{(m-1)} \in \{0,1\}} & \mathbb{P}(O_n^{(1)} = i_n^{(1)} | \mathbf{O}^{(1,n-1)} = \mathbf{i}^{(1,n-1)}) \\ & \times \mathbb{P}(O_n^{(2)} = i_n^{(2)} | \mathbf{O}^{(1,n)} = \mathbf{i}^{(1,n)}, \mathbf{O}^{(2,n-1)} = \mathbf{i}^{(2,n-1)}) \\ & \times ... \\ & \times \mathbb{P}(O_n^{(m)} = i_n^{(m)} | \mathbf{O}^{(m-1,n)} = \mathbf{i}^{(m-1,n)}, \mathbf{O}^{(m,n-1)} = \mathbf{i}^{(m,n-1)}) \\ & = 2e^{-\lambda_1} - \sum_{i^{(2)}, ..., i^{(m-1)} \in \{0,1\}} \mathbb{P}\left(\substack{O_1^{(1)} = 0, O_1^{(2)} = i^{(2)}, ..., \\ O_1^{(m-1)} = i^{(m-1)}, O_1^{(m)} = 0} \right) \\ & := C.\end{aligned} \tag{S27}$$

This leads to

$$\mathbb{P}(\tau_s > n) = C^n.$$

Since $\mathbb{P}(\tau_s > n) < \mathbb{P}(\tau_s > n-1) < 1$ for all $n \geq 2$, we have $C < 1$, showing that $\mathbb{P}(\tau_s > n)$ is an exponential distribution. □

Furthermore, the definition of τ_s implies that

$$\{\tau_1 > n\} \subset \{\tau_s > n\}, \quad \forall n \in \mathbb{N},$$

where τ_1 is the first activation time of node 1. Since

$$\mathbb{P}(\tau_1 > n) = e^{-\lambda_1 n}.$$

This gives

$$\mathbb{P}(\tau_s > n) \geq e^{-\lambda_1 n},$$

meaning that the distribution, $\mathbb{P}(\tau_s > n)$, for any single branch has a uniform lower bound. And we notice that the constant C in Eq. (S27) is monotonically increasing with respect to the size of the ring, m, indicating that the exponent for a branch located at a large ring is smaller than that of a branch located at a small ring. Therefore, we can find an exponential distribution that is a uniform upper bound for every single branch, and the exponent of the upper bound is related to the targeted exponent of nodes and trunks.

3.2 Activity of branches in bursty activity patterns

In this section, we prove that the distribution of the first activation time for every single branch has uniform upper and lower bounds, which are all heavy-tailed.

Similar to Poisson-like activity patterns, for a ring with m nodes, we also select all links except link $(1, m)$ as trunks. The exponent of every single node (trunk) is the same, denoted as α_1 (α_2).

First, we offer the mathematical definition of the heavy-tailed distribution [2].

Definition 5 (Heavy-tailed distribution)**.** *The distribution of a random variable X is said to have a heavy tail if*

$$\lim_{x\to\infty} e^{\lambda x}\mathbb{P}(X > x) = \infty, \quad \forall \lambda > 0.$$

By Definition 4, we propose the definition of a sequence to be heavy-tailed.

Definition 6 (Heavy-tailed sequence)**.** *A non-negative sequence $\{a_n\}_{n=1}^{\infty}$ is said to be heavy-tailed if*

$$\lim_{n\to\infty} e^{\lambda n} a_n = \infty, \quad \forall \lambda > 0.$$

Under the above definitions, we have the following lemmas.

Lemma 1. *For a discrete non-negative random variable X, let* $a_n = \mathbb{P}(X > n)$*, if*

$$\lim_{n\to\infty} \frac{a_n}{a_{n-1}} = 1, \tag{S28}$$

then X is heavy-tailed.

Proof. By Eq. (S28), we have

$$\lim_{n\to\infty} \frac{e^{\lambda n} a_n}{e^{\lambda(n-1)} a_{n-1}} = e^{\lambda} > 1, \quad \forall \lambda > 0.$$

Therefore,

$$\lim_{n\to\infty} e^{\lambda n} a_n = \infty,$$

meaning that the distribution of X is heavy-tailed. □

One straightforward implication is that the power-law distribution is heavy-tailed.

Another lemma shows that a sequence is heavy-tailed when it is lower-bounded by another heavy-tailed sequence,

Lemma 2. *For two non-negative sequences* $\{a_n\}_{n=1}^{\infty}$ *and* $\{b_n\}_{n=1}^{\infty}$ *that satisfy* $a_0 = b_0 = 1$ *and* $a_n \geq b_n$ *for all* $n \geq 1$*, if*

$$\lim_{n\to\infty} \frac{b_n}{b_{n-1}} = 1,$$

then the sequence $\{a_n\}_{n=1}^{\infty}$ *is heavy-tailed.*

Proof. To see this, we assume that there exists $\lambda_0 > 0$ such that $\lim_{n\to\infty} e^{\lambda_0 n} a_n \neq \infty$. Then $\liminf_{n\to\infty} e^{\lambda_0 n} a_n = c < \infty$, which shows there exists a subsequence $\{a_{n_k}\}_{k=1}^{\infty}$ such that the decay of a_{n_k} is exponential, and

$$\ln a_{n_k} := \sum_{i=1}^{n_k} x_i, \quad x_{n_k} = \ln \frac{a_{n_k}}{a_{n_{k-1}}} \to -\lambda_0 \quad \text{as } n_k \to \infty,$$
$$\ln b_{n_k} := \sum_{i=1}^{n_k} y_i, \quad y_{n_k} = \ln \frac{b_{n_k}}{b_{n_{k-1}}} \to 0 \quad \text{as } n_k \to \infty.$$

Thus there exists n_j such that for all $n_k \geq n_j$, $a_{n_k} < b_{n_k}$, in contradiction with the assumption that $a_n \geq b_n$. □

Since

$$\mathbb{P}(\tau_s > n) \geq \mathbb{P}(\tau_1 > n) \approx C n^{-\alpha_1 + 1},$$

with the help of lemmas above, we obtain that $\mathbb{P}(\tau_s > n)$ is a heavy-tailed distribution and is lower bounded by a power-law distribution with an exponent α_1.

We next turn to derive the uniform upper bounds for branches.

Theorem 3. *For a general ring with m nodes, if the exponent of every single node (trunk) is the same, then*

$$A_n \leq \mathbb{P}(\tau_s > n) \leq B_n$$

holds for any m, where the sequences $\{A_n\}_{n=1}^{\infty}$ *and* $\{B_n\}_{n=1}^{\infty}$ *are both heavy-tailed.*

Proof. Similar to Eq. (S26), for each fixed $\mathbf{i}^{(1,n)}, ..., \mathbf{i}^{(m,n)}$, we have

$$\begin{aligned}
\mathbb{P}(\tau_s > n) = & \sum_{\substack{\mathbf{i}^{(1,n-1)} \\ \circ \mathbf{i}^{(m,n-1)} = \mathbf{0}^{(n-1)}}} \sum_{\substack{\mathbf{i}^{(2,n-1)}, ..., \mathbf{i}^{(m-1,n-1)} \\ \in \{0,1\}^n}} \mathbb{P}(\mathbf{O}^{(1,n-1)} = \mathbf{i}^{(1,n-1)}, ..., \mathbf{O}^{(m,n-1)} = \mathbf{i}^{(m,n-1)}) \\
& \left[1 - p_1(\mathbf{i}^{(1,n-1)}, 1) + \sum_{i^{(2)}, ..., i^{(m-1)} \in \{0,1\}} \mathbb{P} \left(\begin{array}{c} O_n^{(1)}=1, O_n^{(2)}=i^{(2)}, \\ ..., \\ O_n^{(m-1)}=i^{(m-1)}, O_n^{(m)}=0 | \\ \mathbf{O}^{(1,n-1)}=\mathbf{i}^{(1,n-1)}, ..., \mathbf{O}^{(m,n-1)}=\mathbf{i}^{(m,n-1)} \end{array} \right) \right] \\
= & \sum_{\substack{\mathbf{i}^{(1,n-1)} \\ \circ \mathbf{i}^{(m,n-1)} = \mathbf{0}^{(n-1)}}} \sum_{\substack{\mathbf{i}^{(2,n-1)}, ..., \mathbf{i}^{(m-1,n-1)} \\ \in \{0,1\}^n}} \mathbb{P}(\mathbf{O}^{(1,n-1)} = \mathbf{i}^{(1,n-1)}, ..., \mathbf{O}^{(m,n-1)} = \mathbf{i}^{(m,n-1)}) \\
& \left[1 - p_1(\mathbf{i}^{(1,n-1)}, 1) + 1 - p_m(\mathbf{i}^{(m,n-1)}, 1) - \sum_{i^{(2)}, ..., i^{(m-1)} \in \{0,1\}} \mathbb{P} \left(\begin{array}{c} O_n^{(1)}=0, O_n^{(2)}=i^{(2)}, \\ ..., \\ O_n^{(m-1)}=i^{(m-1)}, O_n^{(m)}=0 | \\ \mathbf{O}^{(1,n-1)}=\mathbf{i}^{(1,n-1)}, ..., \mathbf{O}^{(m,n-1)}=\mathbf{i}^{(m,n-1)} \end{array} \right) \right]
\end{aligned}$$

Let

$$C_n^{(m)} = \sum_{\substack{\mathbf{i}^{(1,n-1)} \\ \circ \mathbf{i}^{(m,n-1)} = \mathbf{0}^{(n-1)}}} \sum_{\substack{\mathbf{i}^{(2,n-1)}, ..., \mathbf{i}^{(m-1,n-1)} \\ \in \{0,1\}^n}} \mathbb{P}(\mathbf{O}^{(1,n-1)} = \mathbf{i}^{(1,n-1)}, ..., \mathbf{O}^{(m,n-1)} = \mathbf{i}^{(m,n-1)})(1 - p_1(\mathbf{i}^{(1,n-1)}, 1)),$$

Since the statuses of nodes 1 and m are symmetric. We have

$$C_n^{(m)} \leq \mathbb{P}(\tau_s > n) \leq 2C_n^{(m)},$$

Since $\mathbb{P}(\tau_s > n)$ is heavy-tailed, using Lemma 2, we have $2C_n^{(m)}$ is heavy-tailed when n is sufficiently large. Then $\mathbb{P}(\tau_s > n)$ is upper and lower bounded by heavy-tailed distributions. Let $A_n = \min_m C_n^{(m)}$ and $B_n = \max_m 2C_n^{(m)}$, A_n and B_n are also heavy-tailed and are only related to the targeted exponent of nodes and trunks. □

Finally, we discuss the IET distribution of all links. The distribution is represented by a random variable E,

$$\mathbb{P}(E \leq x) = \frac{1}{|\mathcal{E}|} \sum_{i \in \mathcal{E}} \mathbb{P}(E_i \leq x), \tag{S29}$$

where E_i is a random variable representing the IET distribution of link i, $|\mathcal{E}|$ is the number of

links. By Eq. (S29), we have

$$\min_{i \in \mathcal{E}} \mathbb{P}(E_i \leq x) \leq \mathbb{P}(E \leq x) \leq \min_{i \in \mathcal{E}} \mathbb{P}(E_i \leq x),$$

which means the distribution of all links is upper and lower bounded by the distribution of individual links. When the algorithmic IET distribution of every single link is heavy-tailed (exponential), the distribution of all links is also heavy-tailed (exponential).

4 Connection to intercommunication time

A node x is said to be communicating at time t when x is active and at least one of its neighbors is active. In this case, we can count the time interval between two communication events, that is, the intercommunication time (ICT). We use the distribution of the first communication time to analyze the statistical property of ICTs.

We assume a node x has k links (i.e. k neighbors), and the stochastic process of x and these neighbors is denoted as $\{X_n\}_{n\geq 0}$ and $\{N_n^{(1)}\}_{n\geq 0}, ..., \{N_n^{(k)}\}_{n\geq 0}$, respectively. The stopping time for x is

$$\tau_{com}^{(x)} = \min\{\tau_1, ..., \tau_k\},$$

where τ_j ($j = 1, ..., k$) is the first activation time of link j. We would prove the following conclusions: In bursty (Poisson-like) activity patterns, (a) the ICT distribution of node x is upper and lower bounded by power-law (exponential) distributions, and (b) the ICT distribution converges exponentially to the IET distribution as $k \to \infty$.

We first prove part (a). By the definition of the stopping times, we have

$$\{\tau_1 \leq n\} \subset \{\tau_{com}^{(x)} \leq n\} \subset \{\tau_x \leq n\},$$

and part (a) is obtained.

For part (b), without loss of generality, we assume all links have the same IET distribution. We have

$$\begin{aligned}
\mathbb{P}(\tau_{com}^{(x)} > n) &= \mathbb{P}(\min\{\tau_1, ..., \tau_k\} > n) = \mathbb{P}(\tau_1 > n, ..., \tau_k > n) \\
&= \sum_{\mathbf{w}^{(x,n)} \circ (\mathbf{w}^{(1,n)} + ... + \mathbf{w}^{(k,n)}) = \mathbf{0}^{(n)}} \mathbb{P}(\mathbf{X}^{(n)} = \mathbf{w}^{(x,n)}, \mathbf{N}^{(1,n)} = \mathbf{w}^{(1,n)}, ..., \mathbf{N}^{(k,n)} = \mathbf{w}^{(k,n)}) \\
&= \sum_{\mathbf{w}^{(x,n)} = \mathbf{0}^{(n)}} + \sum_{\substack{\mathbf{w}^{(x,n)} \neq \mathbf{0}^{(n)}, \\ \mathbf{w}_x^{(n)} \circ (\mathbf{w}^{(1,n)} + ... + \mathbf{w}^{(k,n)}) = \mathbf{0}^{(n)}}} .
\end{aligned}$$

The first part of the above equation equals $\mathbb{P}(\tau_x > n)$. We turn to prove that the second part converges exponentially to 0 as $k \to \infty$.

For any trajectory $\mathbf{w}^{(x,n)} \neq \mathbf{0}^{(n)}$, node x is activated at least once during time 1 to n, we assume the last activation time is $m \leq n$. For any tuple $(\mathbf{w}^{(x,n)}, \mathbf{w}^{(1,n)}, ..., \mathbf{w}^{(k,n)})$ satisfies the condition of the second part, we have

$$\begin{aligned}
\mathbb{P}(\mathbf{X}^{(n)} = \mathbf{w}^{(x,n)}, ..., \mathbf{N}^{(k,n)} = \mathbf{w}^{(k,n)}) &\leq \mathbb{P}(\mathbf{X}^{(m)} = \mathbf{w}^{(x,m)}, ..., \mathbf{N}^{(k,m)} = \mathbf{w}^{(k,m)}) \\
&= \mathbb{P}(\mathbf{X}^{(m-1)} = \mathbf{w}^{(x,m-1)}, ..., \mathbf{N}^{(k,m-1)} = \mathbf{w}^{(k,m-1)}) \\
&\times \mathbb{P}\left(\substack{\mathbf{X}_m = 1, \mathbf{N}_m^{(1)} = 0, ..., \mathbf{N}_m^{(k)} = 0 | \\ \mathbf{X}^{(m-1)} = \mathbf{w}^{(x,m-1)}, ..., \mathbf{N}^{(k,m-1)} = \mathbf{w}^{(k,m-1)}}\right) \\
&= \mathbb{P}(\mathbf{X}^{(m-1)} = \mathbf{w}^{(x,m-1)}, ..., \mathbf{N}^{(k,m-1)} = \mathbf{w}^{(k,m-1)}) \\
&\times \mathbb{P}(\mathbf{X}_m = 1 | \mathbf{X}^{(m-1)} = \mathbf{w}^{(x,m-1)}) \\
&\times \mathbb{P}(\mathbf{N}_m^{(1)} = 0 | \mathbf{X}^{(m)} = \mathbf{w}^{(x,m)}, \mathbf{N}^{(1,m-1)} = \mathbf{w}^{(1,m-1)}) \\
&... \\
&\times \mathbb{P}(\mathbf{N}_m^{(k)} = 0 | \mathbf{X}^{(m)} = \mathbf{w}^{(x,m)}, \mathbf{N}^{(k,m-1)} = \mathbf{w}^{(k,m-1)}). \\
&\leq p_x(\mathbf{w}^{(x,m-1)}, 1) \left(\frac{p_x(\mathbf{w}^{(x,m-1)}, 1) - p_z(\mathbf{0}^{(m-1)}, 1)}{p_x(\mathbf{w}^{(x,m-1)}, 1)} \right)^k
\end{aligned},$$

where $p_z(\mathbf{0}^{(m-1)}, 1)$ represents the conditional probability of links. As the number of the tuple $(\mathbf{w}^{(x,n)}, \mathbf{w}^{(1,n)}, ..., \mathbf{w}^{(k,n)})$ satisfying the condition of the second part is finite and

$$\frac{p_x(\mathbf{w}^{(x,m-1)}, 1) - p_z(\mathbf{0}^{(m-1)}, 1)}{p_x(\mathbf{w}^{(x,m-1)}, 1)} < 1,$$

the second part decays exponentially to 0.

When the average degree of underlying topologies is sufficiently large, the ICT distribution of nodes is almost the same as the IET distribution.

5 Statistical test in empirical datasets

In our theoretical model, we assume that the activity of every single node and trunk follows a renewal process and the activity of nodes is somehow conditionally independent (Eq. (S4)). Here we use statistical inference to verify whether the above two assumptions hold in empirical datasets.

For the first assumption, we need to demonstrate that the IET samples of single nodes and links come from the same distribution and are independent of each other. We begin with counting the IET samples of each node/link. For each node/link with n samples, we randomly divide them into two sets of size $n/2$. We use the Kolmogorov–Smirnov test [4] to verify whether these two sets are from the same distribution, which coincides with the null hypotheses. For the conditional independence, we use the Spearman rank correlation test [5],

in which the null hypothesis is that the two sets are uncorrelated.

Supplementary Fig. 6 shows that the null hypotheses are accepted in both the Spearman rank correlation test and the Kolmogorov–Smirnov test, meaning that the activity of nodes and links is a renewal process in empirical datasets. Furthermore, the distributions of the p-value for empirical datasets match with that for the corresponding synthetic temporal networks. Another important finding is that although we have proved that the activity of branches is not a strict renewal process, most of the branches still pass the tests. This indicates that the approximation we have done in Section 3 is reasonable.

For the second assumption, we cannot directly calculate whether the left-hand side of Eq. (S4) equals the right-hand side through statistics since we only have one trajectory for each node and link in empirical datasets. We offer an alternative solution to test the conditional independence in Eq. (S4). Specifically, for each pair of nodes in the underlying topology, we count the conditional IET samples of one node x when the other node y is active or inactive, which forms two sets of samples. Then we use the Kolmogorov–Smirnov test over these two sets. If the null hypothesis is valid, Eq. (S4) holds in empirical datasets. This is because the relation

$$\begin{aligned}&\mathbb{P}(X_{m+k}=1,...,X_{m+1}=0|X_m=1,\mathbf{X}^{(n-1)}=\mathbf{x}^{(n-1)},\mathbf{Y}^{(n)}=\mathbf{y}^{(n)})\\&=\mathbb{P}(X_k=1,...,X_1=0)\end{aligned}\tag{S30}$$

holds for any m, k under Equation (S4). It is straightforward to check the left-hand side of Eq. (30) equals

$$\frac{\mathbb{P}(X_{m+k}=1,...,X_{m+1}=0,X_m=1,\mathbf{X}^{(n-1)}=\mathbf{x}^{(n-1)},\mathbf{Y}^{(n)}=\mathbf{y}^{(n)})}{\mathbb{P}(X_m=1,\mathbf{X}^{(n-1)}=\mathbf{x}^{(n-1)},\mathbf{Y}^{(n)}=\mathbf{y}^{(n)})},$$

and the numerator equals

$$\begin{aligned}&\sum_{y_i\in\{0,1\},\ i=1,...,k-1}\mathbb{P}(X_{m+k}=1,Y_{m+k-1}=y_{m+k-1},...,X_{m+1}=0,Y_{m+1}=y_1,\\&X_m=1,\mathbf{X}^{(n-1)}=\mathbf{x}^{(n-1)},\mathbf{Y}^{(n)}=\mathbf{y}^{(n)})\\&=\mathbb{P}(X_k=1|X_{k-1}=0,...,X_1=0)\mathbb{P}(X_{m+k-1}=0,...,X_m=1,\mathbf{X}^{(n-1)}=\mathbf{x}^{(n-1)},\mathbf{Y}^{(n)}=\mathbf{y}^{(n)})\\&=\mathbb{P}(X_k=1,...,X_1=0)\mathbb{P}(X_m=1,\mathbf{X}^{(n-1)}=\mathbf{x}^{(n-1)},\mathbf{Y}^{(n)}=\mathbf{y}^{(n)}).\end{aligned}$$

Therefore, the left-hand side equals the right-hand side of Eq. (S30). Supplementary Fig. 7 shows the corresponding statistical results. The distribution of p-values indicates that Eq. (S4) does not hold in empirical datasets.

Nevertheless, we still find weaker conditional independence in empirical datasets. Specifically, we use the Chi-squared test [6] to validate if the following relation holds

$$\begin{aligned}&\mathbb{P}(X_{n+1}=1|X_n=0,...,X_{n-l+2}=0,X_{n-l+1}=1,Y_n=0)\\&=\mathbb{P}(X_{n+1}=1|X_n=0,...,X_{n-l+2}=0,X_{n-l+1}=1,Y_n=1)\end{aligned}\tag{S31}$$

Algorithm 4 Combination with network evolution

Input: initial underlying topology $\mathcal{G}$ and parameter t_{tol}
Output: trajectories of all nodes and links

1: Select a spanning tree $\mathcal{T}$ of $\mathcal{G}$
2: Assign a probability mass function to each node and trunk in $\mathcal{T}$
3: **for** $t = 1$ to t_{tol} **do**
4: Execute a single loop of Algorithm 3 on $\mathcal{G}$
5: **if** $\mathcal{G}$ evolves **then**
6: Update $\mathcal{G}$ by network evolution
7: Update $\mathcal{T}$ on the new underlying topology
8: Assign a targeted distribution to each new node/trunk and set them to be active
9: Update the state of old nodes and links
return trajectories of all nodes and links

The parameter l represents the IET from the last activation time. If Eq. (S4) holds, the null hypotheses for the test should be valid for any possible l. We find that the null hypotheses become more easily accepted when l is larger (fig. S8). This means that the state of y matters only when x tries to activate frequently.

6 Time-varying underlying topologies

Another pivotal advantage of our algorithm is easily integrated with the evolution of networks. Algorithm 4 demonstrates a unified framework to construct temporal networks with evolving underlying topologies.

The most common network evolution model is the network growth model, in which new nodes with links sequentially enter a network system and connect to old nodes [7] (including the example presented in the main text). In addition, when considering recessionary effects, the number of nodes and links may decrease [8]. For different network evolution, the main design in Algorithm 4 is how to update $\mathcal{T}$. A reasonable design is necessary when nodes and links may vanish on the underlying topology during the evolution.

7 Analysis of aggregated networks

In addition to studying the IET distribution, we analyze the structural measures of aggregated networks (fig. S11) with different aggregation times t_{agg} to evaluate other temporal properties of our synthetic temporal networks. We study two typical measures, the expected number of activations of individual nodes (links) and the node strength distribution of aggregated networks. The former is a micro statistic capturing the frequency of activations of each unit in networks, and the latter is a macro statistic showing the structural information

of entire networks.

For a node i (respectively trunk j) whose targeted IET distribution is $\phi_i(\Delta t, \alpha_i)$ (respectively $\psi_j(\Delta t, \beta_j)$), the expectation of ϕ_i (respectively ψ_j) is denoted as $\mu(\alpha_i)$ (respectively $v(\beta_j)$), where the exponent α_i (respectively β_j) is obtained by sampling from a distribution η_{node} (respectively η_{link}). The total activation number of node i up to moment t is denoted as $A_t^{(i)}$. Let

$$A^{(i)}(t) = \mathbb{E}A_t^{(i)}.$$

Using the elementary renewal theorem [3], we have

$$\frac{A^{(i)}(t)}{t} \to \frac{1}{\mu(\alpha_i)} \quad \text{as } t \to \infty, \tag{S32}$$

where $\frac{1}{\infty} = 0$. The conclusion for single links is similar. Equation (S32) presents an intuitive conclusion that the average growth rate of activation numbers asymptotically equals the frequency of activations. In a bursty activity pattern, when $\alpha < 2$, the expectation $\mu(\alpha) = \infty$. This suggests that the growth is sublinear, and the rate asymptotically equals 0. In a Poisson-like activity pattern, for any exponent α, $\mu(\alpha) < \infty$, thus the growth is linear.

Another important statistic for an aggregated network is the node strength distribution. In empirical datasets, we find that the node strength distributions present specific robustness across different time scales (fig. S12). Here we prove that our model also reproduces this property.

For a static unweight network $\mathcal{G}$, the degree distribution of $\mathcal{G}$ is denoted as $d(x)$ and its maximum value is k_{max}. We set a random variable X of which the probability mass function is $d(x)$. Let N_t denote the strength of a node in the aggregated network generated by $\mathcal{G}$ with the aggregation time, $t_{agg} = t$, N_t is a random variable. We assume that the exponent of all links is sampled from η_{link}, using a limit theorem of renewal theory [3], with probability 1,

$$\frac{N_t}{t} \to \sum_{i=1}^{X} \frac{1}{v(\eta_i)} \quad \text{as } t \to \infty, \tag{S33}$$

where $\{\eta_i\}_{1 \le i \le k_{max}}$ is a sequence of independent random variables with a common distribution η_{link}.

As X and $\{\eta_i\}_{1 \le i \le k_{max}}$ are independent, when t is sufficiently large, from Eq. (S33), the distribution of N_t is given by

$$\mathbb{P}(N_t \le s) = \sum_{x=1}^{k_{max}} \mathbb{P}(X = x) F^{(x)}(\frac{s}{t}), \tag{S34}$$

where $F^{(x)}$ is the x-order convolution of the distribution function of the random variable $1/v(\eta_1)$. The Laplace transform of the random variable X is defined by $\rho_X(s) = \mathbb{E}e^{-sX}$, then

Eq. (S34) is converted into

$$\rho_{N_t}(s) = \sum_{x=1}^{k_{max}} \mathbb{P}(X = x)[\rho_{\frac{1}{v(\eta_1)}}(st)]^x.$$

In particular, when a.s. η_i is a constant and equals β, Eq. (S34) can be estimated as follows

$$\mathbb{P}(N_t \leq s) \approx \int_0^{sv(\beta)/t} d(x)\mathrm{d}x. \tag{S35}$$

Formally, let $p_{N_t}(x)$ denote the probability density function of N_t. From Eq. (S35), we have

$$p_{N_t}(x) = \frac{v(\beta)d(\frac{xv(\beta)}{t})}{t}. \tag{S36}$$

Equation (S36) shows the relationship between the degree distribution of a static network and the node strength distribution of the aggregated network. When $d(x) = Cx^{-\gamma}$, the node strength distribution is also power-law with the same exponent γ for each aggregation time, indicating the robustness of aggregated networks. Figure 5a shows the survival function of node strength with different t_{agg} based on scale-free underlying topologies. As one can see, the results are all power-law distributions with the same exponent as the degree distribution, which suggests that the distribution of node strength is robust to the aggregation time and the given distribution.

For general degree distributions, we can also obtain a similar robust behaviour of node strength distributions. For two aggregated networks G_1, G_2 with the aggregation time t_1, t_2 ($t_1 > t_2$), we have

$$\mathbb{P}(Xt_2/v(\beta) > s) = F^{(2)}(s) = F^{(1)}(s\frac{t_1}{t_2}) := F^{(1)}(\hat{s}),$$

where $F^{(i)}(s)$ is the survivor function of the random variable $Xt_i/v(\beta)$ ($i = 1, 2$). When s in $F^{(2)}$ changes from s_1 to $s_1 + 1$, $\hat{s}$ in $F^{(1)}$ changes from $s_1\frac{t_1}{t_2}$ to $(s_1 + 1)\frac{t_1}{t_2}$, which means the proportion of nodes with strength between $s_1\frac{t_1}{t_2}$ and $(s_1 + 1)\frac{t_1}{t_2}$ in G_1 is same as the proportion of nodes with strength s_1 in G_2.

The normalization is executed as follows. We first select a sufficiently large moment, t_{base}, and its corresponding aggregated network, G_{base}, is said to be the baseline. For any aggregation time $t_{agg} \geq t_{base}$, when all links have the same targeted distribution, the proportion of nodes with strength between st_{agg}/t_{base} and $(s+1)t_{agg}/t_{base}$ in G_{agg} is same as that with strength s in G_{base}. Therefore, the normalized distribution of node strength for the aggregated network $G_{t_{agg}}$ is the same as that for $G_{t_{base}}$. We verify the above robustness on the small-world underlying topology under different activity patterns (Fig. 5b). The normalized distribution for different aggregation times all collapses onto the node strength of the baseline network.

8 Statistics for measuring burstiness and temporal correlations

The burstiness parameter B is widely used to measure the level of burstiness [9], defined by the coefficient of variation,

$$B = \frac{\sigma/\mu - 1}{\sigma/\mu + 1},$$

where μ and σ are the mean and standard deviation of IET distributions. When μ and σ are finite, the definition is meaningful and $|B| < 1$.

We calculate the burstiness parameter of nodes and links for the synthetic temporal networks in Fig. 2. In order to compare algorithmic and theoretical burstiness parameters, we set a cutoff κ for theoretical calculation. The theoretical mean μ and standard deviation σ for a power-law distribution are

$$\mu = \frac{\sum_{i=1}^{\kappa} i^{-\gamma+1}}{\sum_{i=1}^{\kappa} i^{-\gamma}}, \quad \sigma = \left(\frac{\sum_{i=1}^{\kappa} i^{-\gamma+2}}{\sum_{i=1}^{\kappa} i^{-\gamma}} - \mu^2 \right)^{\frac{1}{2}},$$

and for a discrete exponential distribution are

$$\mu = e^{\gamma/2} \sum_{i=1}^{\kappa} i (e^{-\gamma(i-0.5)} - e^{-\gamma(i+0.5)}), \quad \sigma = \left(e^{\gamma/2} \sum_{i=1}^{\kappa} i^2 (e^{-\gamma(i-0.5)} - e^{-\gamma(i+0.5)}) - \mu^2 \right)^{\frac{1}{2}}.$$

Tables S1 and S2 compare the simulation and theoretical B in bursty activity patterns and in Poisson-like activity patterns, respectively. The simulations are robust to underlying topologies and are well-predicted by theoretical results. Both nodes and links show a high level of burstiness in bursty activity patterns and present a negative (low) level in Poisson-like activity patterns.

We also investigate the temporal correlation of synthetic temporal networks. The autocorrelation function $A(\Delta t)$ is a common statistic to appreciate the global activity correlations for temporal networks. For a temporal network $\mathcal{G} = \{G_1, ..., G_T\}$, the autocorrelation coefficient $A(\Delta t)$ is defined as

$$A(\Delta t) = \frac{\frac{1}{T-\Delta t} \sum_{i=1}^{T-\Delta t} E(i)E(i+\Delta t) - \mu_1\mu_2}{\sigma_1\sigma_2},$$

where $E(i)$ denotes the total activation numbers of nodes or links in the snapshot i, μ_1, σ_1^2 (respectively μ_2, σ_2^2) are the sample mean and sample variance of the series $\{E(i)\}_{i=1}^{T-\Delta t}$ (respectively $\{E(i+\Delta t)\}_{i=1}^{T-\Delta t}$). The parameter Δt represents the distance of the time windows in the two series. In particular, when $\Delta t = 1$, $A(\Delta t)$ is called the memory coefficient [9].

By Hölder's inequality, we obtain that $|A(\Delta t)| \leq 1$. The closer $A(\Delta t)$ is to 0, the less correlated $\{E(i)\}_{i=1}^{T-\Delta t}$ and $\{E(i+\Delta t)\}_{i=1}^{T-\Delta t}$, and the weaker the autocorrelation of the temporal network. When $A(\Delta t)$ is close to 1 or -1, A strong positive or negative linear correlation exists

between series $\{E(i) - \mu_1\}_{i=1}^{T-\Delta t}$ and series $\{E(i+\Delta t) - \mu_2\}_{i=1}^{T-\Delta t}$.

Supplementary Fig. 14 shows the results of $A(\Delta t)$ in bursty activity patterns and Poisson-like activity patterns. In bursty activity patterns, the construction process is a non-Markovian process, and the results of $A(\Delta t)$ show positive temporal correlations for all time intervals Δt, which is consistent with heterogeneous temporal behaviour discovered in empirical temporal networks. In Poisson-like activity patterns, the results of $A(\Delta t)$ are almost 0. Actually, Eq. (S10) indicates that the activity of single nodes/trunks is a discrete-time Markov chain $\{M_n\}_{n\geq 0}$ with two states $\{s_0, s_1\}$, where s_0 represents the element (node or trunk) is inactive and s_1 represents the element is active. We set $s_0 = 0$ and $s_1 = 1$. The corresponding transition probability matrix is given by follows

$$\begin{array}{cc} & \begin{array}{cc} s_0 & s_1 \end{array} \\ \begin{array}{c} s_0 \\ s_1 \end{array} & \begin{pmatrix} e^{-\lambda} & 1-e^{-\lambda} \\ e^{-\lambda} & 1-e^{-\lambda} \end{pmatrix} \end{array},$$

where λ is the exponent of the targeted distribution. For all $m > 1$, we have

$$\begin{aligned} \mathbb{P}(M_m = 1) &= \sum_{i_1,\ldots,i_{m-1}\in\{0,1\}} \mathbb{P}(M_1 = i_1, \ldots, M_{m-1} = i_{m-1}, M_m = 1) \\ &= \sum_{i_1,\ldots,i_{m-1}\in\{0,1\}} \mathbb{P}(M_1 = i_1, \ldots, M_{m-1} = i_{m-1}) \mathbb{P}(M_m = 1 | M_{m-1} = i_{m-1}) \\ &= 1 - e^{-\lambda}. \end{aligned}$$

Hence, for all $n, m \geq 1$, we have

$$\begin{aligned} \mathbb{P}(M_n = 1, M_{n+m} = 1) &= \sum_{i_1,\ldots,i_{m-1}\in\{0,1\}} \mathbb{P}(M_n = 1, M_{n+1} = i_1, \ldots, M_{n+m-1} = i_{m-1}, M_{n+m} = 1) \\ &= \sum_{i_1,\ldots,i_{m-1}\in\{0,1\}} \mathbb{P}(M_n = 1, \ldots, M_{n+m-1} = i_{m-1}) \mathbb{P}(M_{n+m} = 1 | M_{n+m-1} = i_{m-1}) \\ &= (1 - e^{-\lambda})^2. \end{aligned}$$

This gives

$$\begin{aligned} A(\Delta t) &\sim \frac{1}{T-\Delta t} \sum_{i=1}^{T-\Delta t} \mathbb{E} M_i M_{i+\Delta t} - \frac{1}{(T-\Delta t)^2} \sum_{i,j=1}^{T-\Delta t} \mathbb{E} M_i \mathbb{E} M_{i+\Delta t} \\ &= \frac{1}{T-\Delta t} \sum_{i=1}^{T-\Delta t} \mathbb{P}(M_i = 1, M_{i+\Delta t} = 1) - \frac{1}{(T-\Delta t)^2} \sum_{i,j=1}^{T-\Delta t} \mathbb{P}(M_i = 1) \mathbb{P}(M_{i+\Delta t} = 1) \\ &= 0, \end{aligned}$$

meaning that Poisson-like activity patterns are memoryless (or homogenous).

Supplementary Table 1: Burstiness parameter of bursty activity patterns. The cutoff κ is set to be 1×10^4. The exponent of the simulation is the fitted exponent of algorithmic distributions, and the exponent of the theory is set to the average of the two simulation exponents.

Dataset	Object	Exponent	B	Object	Exponent	B
Simulation (BA)	Nodes	1.80	0.82	Links	1.28	0.64
Simulation (SW)	Nodes	1.80	0.82	Links	1.28	0.64
Theory	Nodes	1.80	0.84	Links	1.28	0.60
Simulation (BA)	Nodes	2.00	0.83	Links	1.69	0.80
Simulation (SW)	Nodes	2.00	0.83	Links	1.71	0.79
Theory	Nodes	2.00	0.86	Links	1.70	0.81

Supplementary Table 2: Burstiness parameter of Poisson-like activity patterns. The cutoff κ is set to be 10^3. The implication of parameters is the same as Table 1.

Dataset	Object	Exponent	B	Object	Exponent	B
Simulation (BA)	Nodes	1.80	-0.42	Links	1.21	-0.29
Simulation (SW)	Nodes	1.80	-0.42	Links	1.22	-0.29
Theory	Nodes	1.80	-0.42	Links	1.21	-0.29
Simulation (BA)	Nodes	2.50	-0.55	Links	1.86	-0.43
Simulation (SW)	Nodes	2.50	-0.55	Links	1.89	-0.44
Theory	Nodes	2.50	-0.55	Links	1.87	-0.43

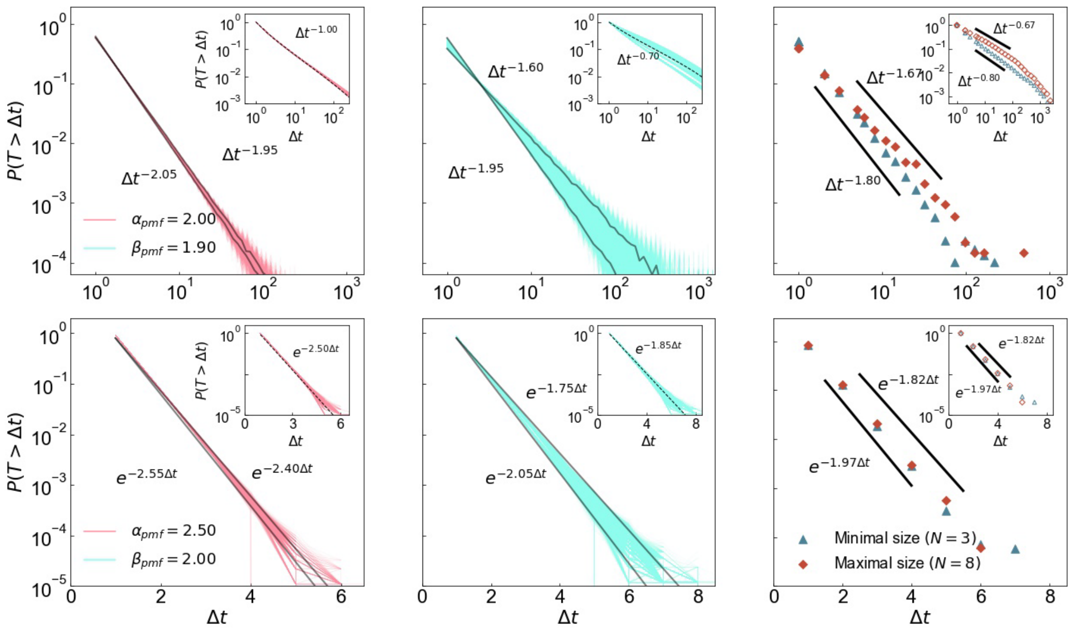

Supplementary Figure 1: Single IET distributions of every element. The algorithmic IET distribution of every single node (link) is represented by a red (green) line, respectively, showing in the first and second columns. The black lines are the upper or lower bounds of the algorithmic results, which are power-law distributions in the first row and exponential distributions in the second row generated by simulations. We also select the results of branches located in the largest and smallest ring showing in the third column by diamonds and triangles. The branch located in the largest ring has a smaller fitted exponent than that in the smallest ring, which is consistent with the theoretical result.

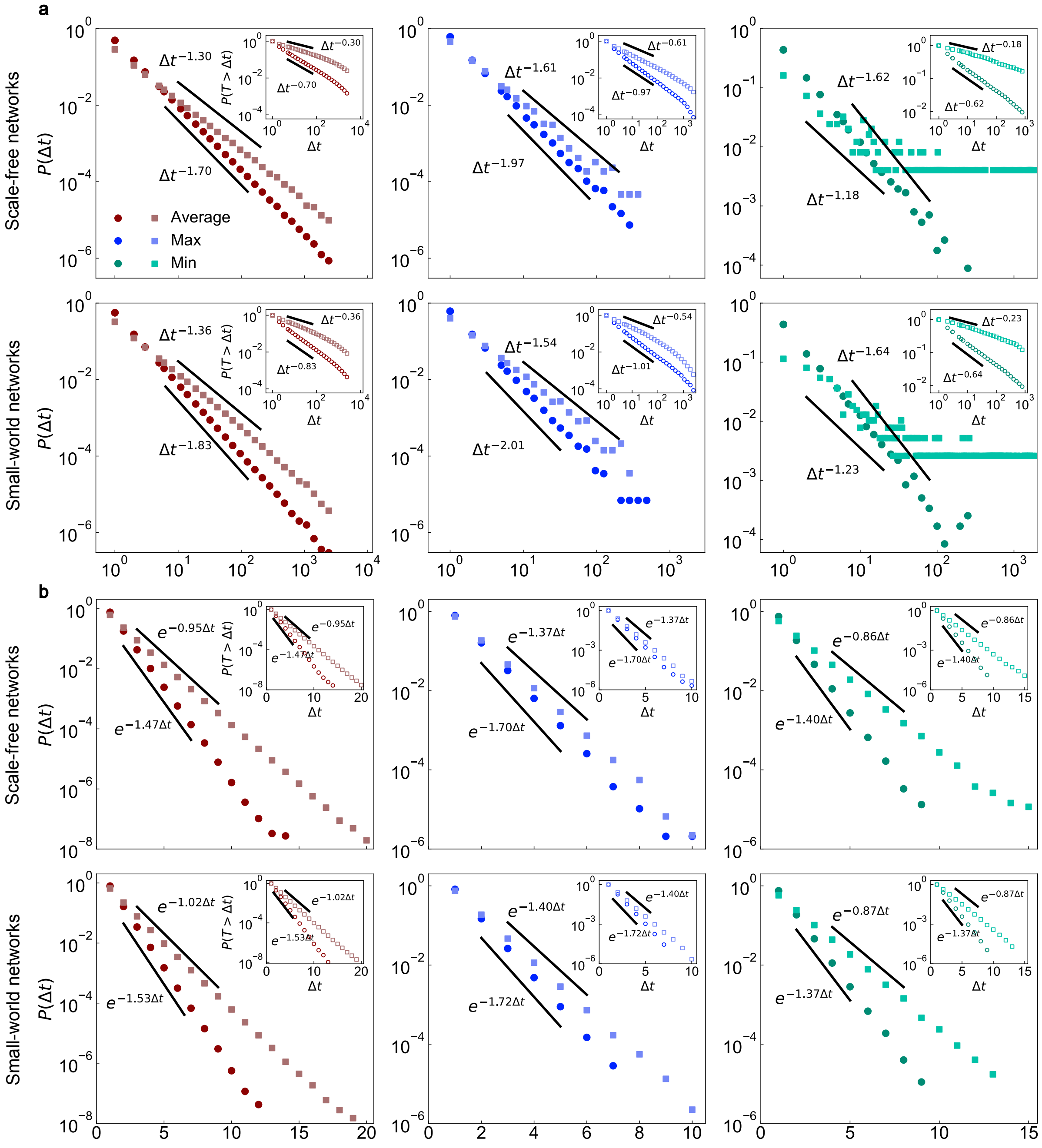

Supplementary Figure 2: IET distributions when exponents are sampled from distributions. We consider the case that the exponent of every single node (trunk) is a random variable η_{node} (η_{link}). We set η_{node} and η_{link} to a uniform $[1.60, 2.00]$ random variable and a uniform $[1.20, 1.50]$ random variable in the bursty activity pattern (**a**) and a uniform $[1.40, 1.70]$ random variable and a uniform $[1.05, 1.30]$ random variable in the Poisson-like activity pattern (**b**). The results of nodes (links) are represented by circles (squares). The aggregated distribution of nodes/links is plotted by brown markers, and the single distribution of the node/link with the maximal (minimal) activation numbers is plotted by blue (green) markers. Parameter values are the same as those in Fig. 2.

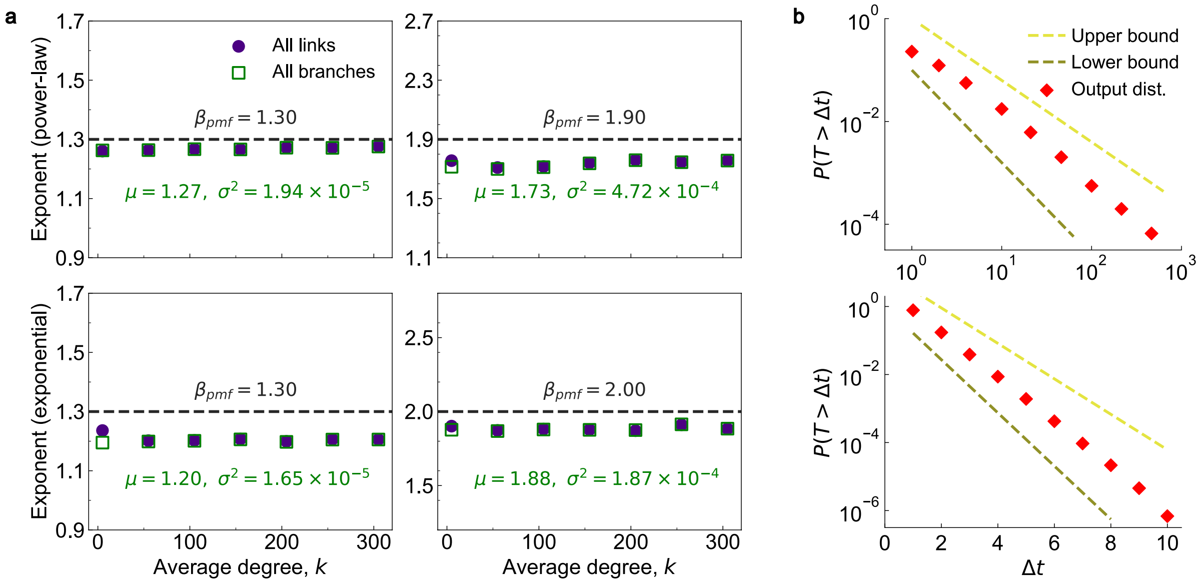

Supplementary Figure 3: Aggregated IET distribution of links is stable in different underlying topologies. **a** Relationship between the underlying topology and the algorithmic exponent. Random regular graphs with different average degrees, k, are utilized to represent different underlying topologies. We plot the algorithmic exponent for all branches (hollow squares) and all links (i.e. all trunks and branches, solid circles). Black dashed lines show the targeted exponent for trunks. When the average degree becomes larger, the proportion of branches in all links increases. As a result, the algorithmic exponent for all links gradually converges to that for all branches. Furthermore, the algorithmic exponents do not correlate significantly with k. The largest magnitude of variances is no more than 10^{-3}, indicating the robustness to the underlying topology. Parameter value: the size of underlying topologies $N = 400$. **b** Schematic illustration of the upper and lower bounds for individual branches. The algorithmic distribution of every single branch (red diamonds) has uniform upper and lower bounds, which are heavy-tailed distributions in a bursty activity pattern (top panel) and exponential distributions in a Poisson-like activity pattern (bottom panel).

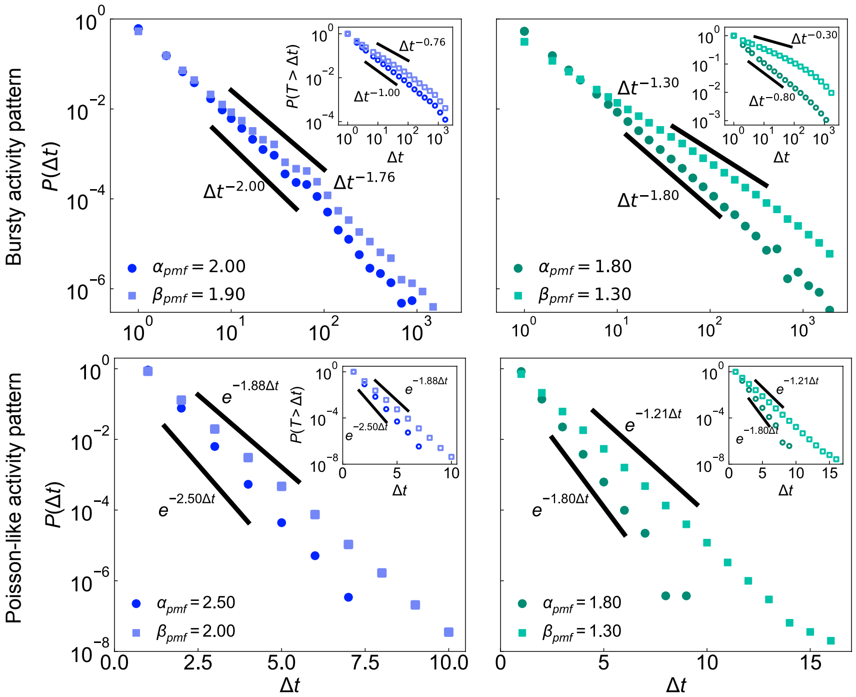

Supplementary Figure 4: IET distributions on well-mixed networks. The largest relative deviation of the algorithmic exponent between Fig. 2 and this is $2 \times |1.76 - 1.69| / (1.76 + 1.69) \approx 4\%$. Parameter value: the size of underlying topologies $N = 400$. Other parameter values are the same as in Fig. 2.

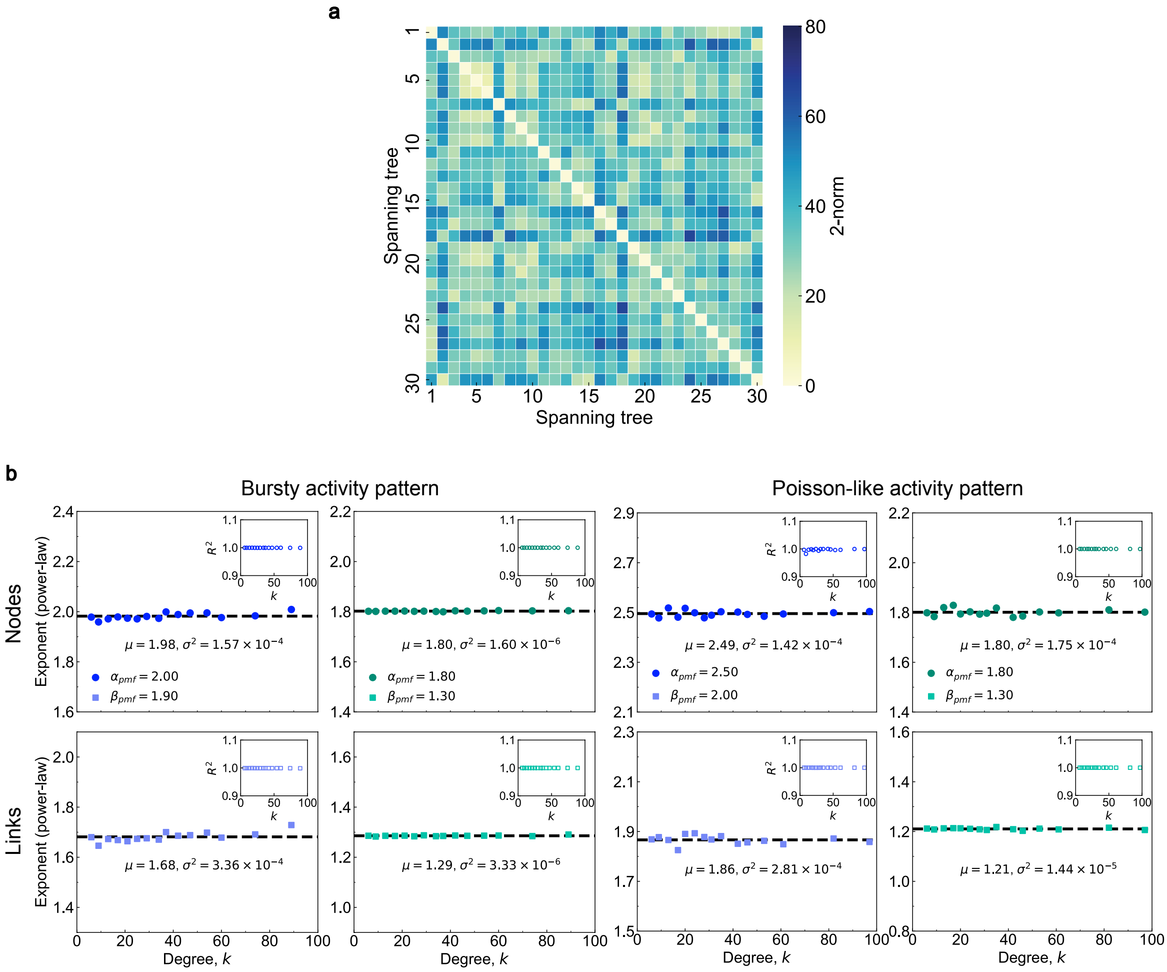

Supplementary Figure 5: Aggregated IET distribution of links is stable for different spanning tree selections. **a** The underlying topology is the Barabási-Albert scale-free network. Nodes with different degrees are selected to be the root of a spanning tree to represent the difference in spanning tree selection. To check whether there is an isomorphism between a pair of spanning trees, we first calculate their ascended node degree sequences and calculate the 2-norm of the difference of their node degree sequences. The result shows that the topology of all spanning trees is different (i.e. not isomorphic). **b** The algorithmic fitted exponent does not correlate significantly with the selection of spanning trees under both patterns, and the largest magnitude of variances is no more than 10^{-4}, indicating the robustness to spanning tree selection.

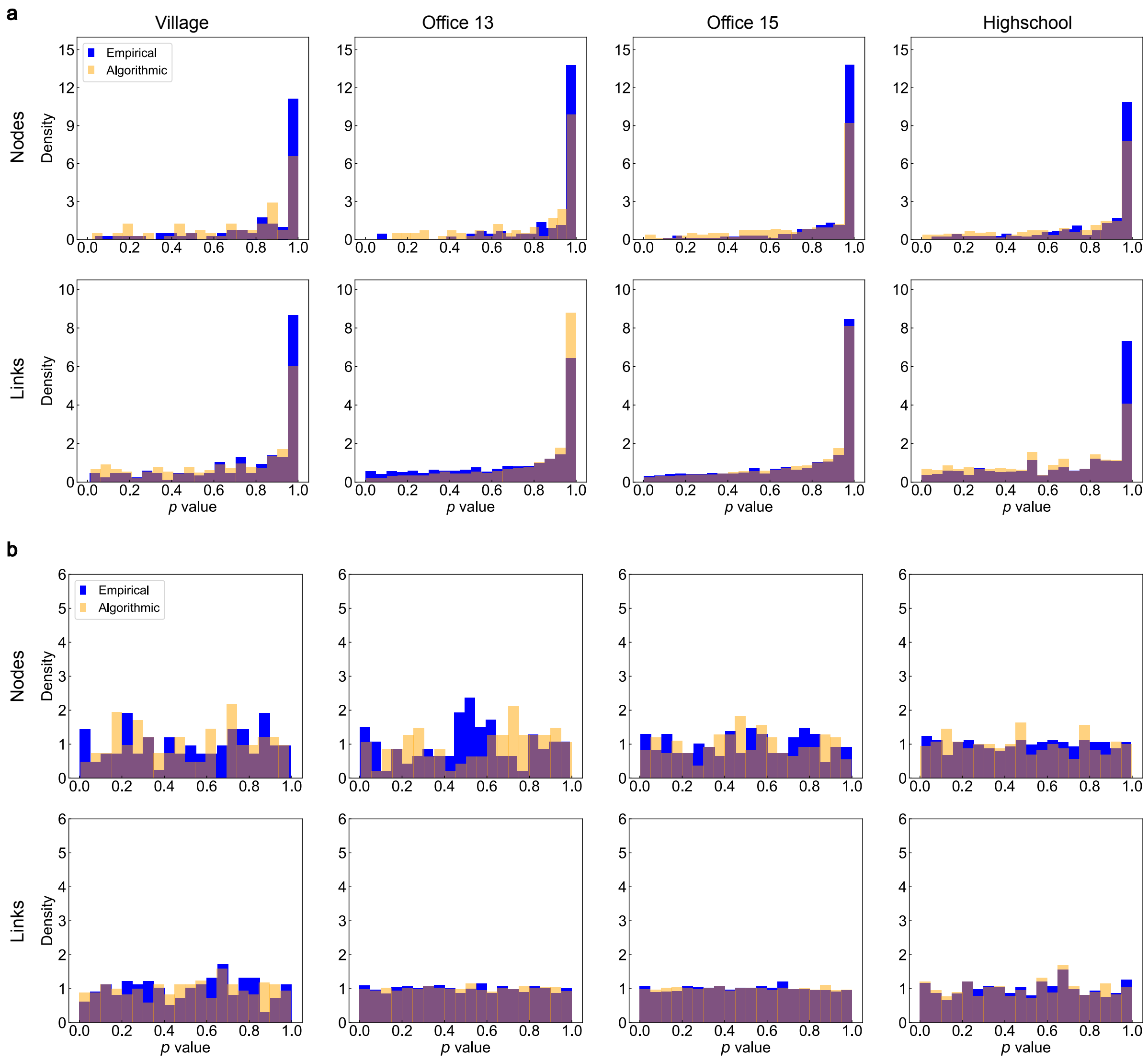

Supplementary Figure 6: Empirical evidence of the activity of nodes/links being renewal processes. We consider the statistical inference for the assumptions that the IET samples of each node/edge come from the same distribution (**a**) and are independent of each other (**b**). For each node/link, we first obtain all its IET samples and randomly divide them into two sets. For (**a**), we use the K-S test to judge whether these two sets are from the same distribution. For (**b**), we use the Spearman rank correlation test to judge whether these two sets are independent. We obtain the p-values of nodes and links with more than 6 IET samples and display them as histograms. Blue (yellow) bars show the distribution of the p-value for empirical (synthetic) datasets.

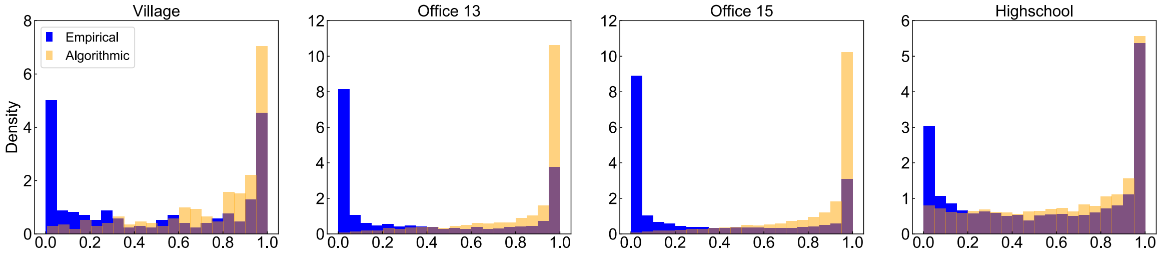

Supplementary Figure 7: Testing Eq. (S30) in empirical datasets. For each pair of nodes, we use the K-S test to judge whether the IET distribution of one node remains identical when the current state of the other node is active or inactive. The synthetic temporal networks definitely can pass the test, but the empirical datasets reject the conditional independence in Eq. (S30).

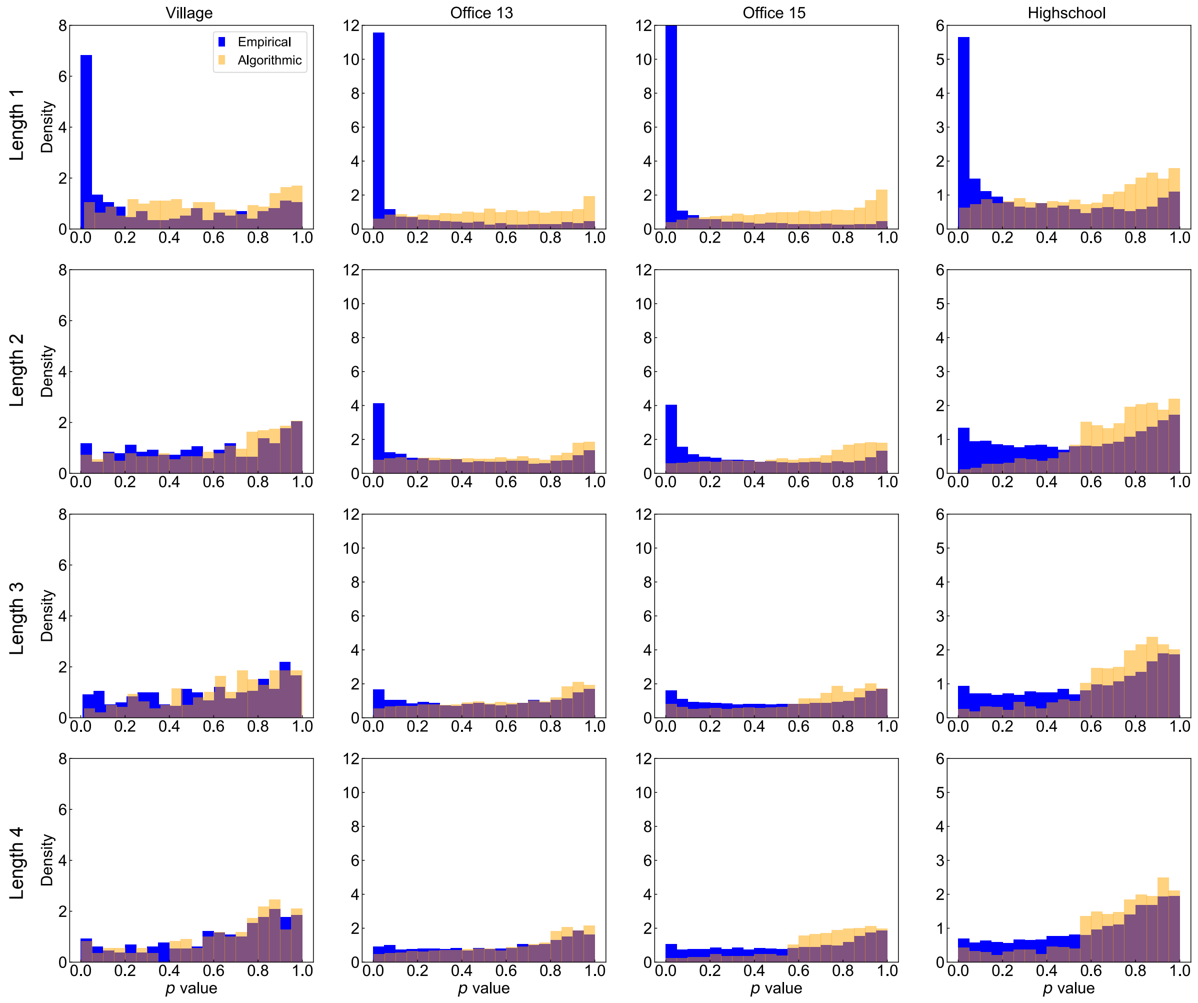

Supplementary Figure 8: Conditional independence tests under different memory lengths. We test Eq. (S31) with the Chi-squared test under different lengths l. When l becomes larger, the distributions of $p-$values in the empirical datasets is more closed to that in the corresponding synthetic temporal networks. Therefore, the conditional independence under a large length (such as $l = 3, 4$) is valid.

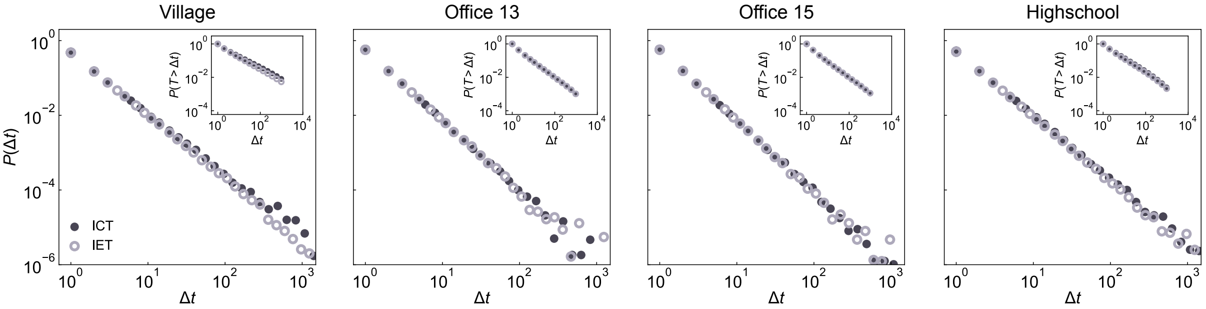

Supplementary Figure 9: Comparison between the IET and ICT distributions of nodes. The average degree of the underlying topologies for these datasets is 8.24, 82.42, 152.74, and 35.84, respectively, showing a high level of connectivity among populations. Therefore, the ICT distributions collapse into the IET distributions.

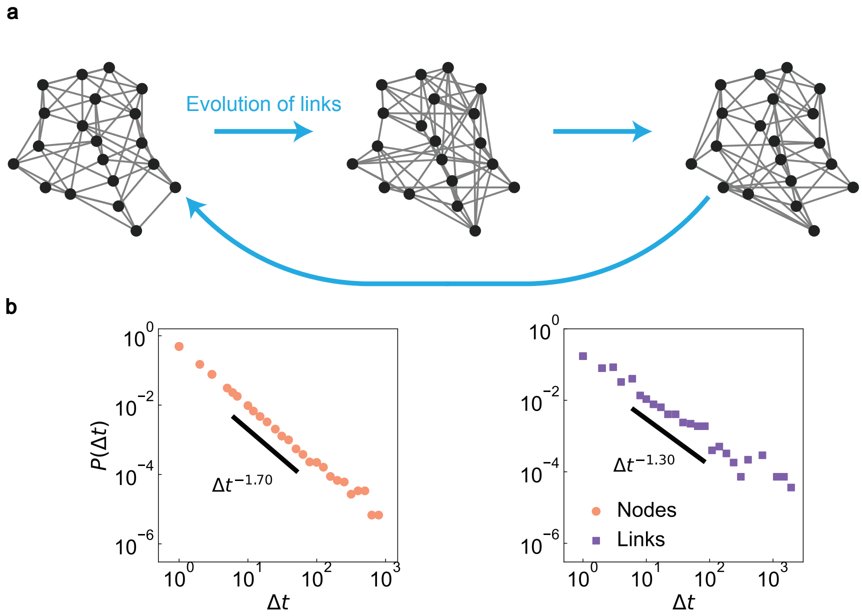

Supplementary Figure 10: Burstiness on a time-varying underlying topology with periodic transition. **a** We consider a time-varying underlying topology with periodic transition. The three networks are the topologies at the corresponding time steps and are all small-world networks of size $N = 20$ and average degree $k = 6$. **b** We generate the bursty activity pattern of node exponent $\alpha = 1.7$ and edge exponent $\beta = 1.3$ on this time-varying topology. The result shows that our algorithm is also applicable for this case. Parameter: the length of temporal network $T = 3000$.

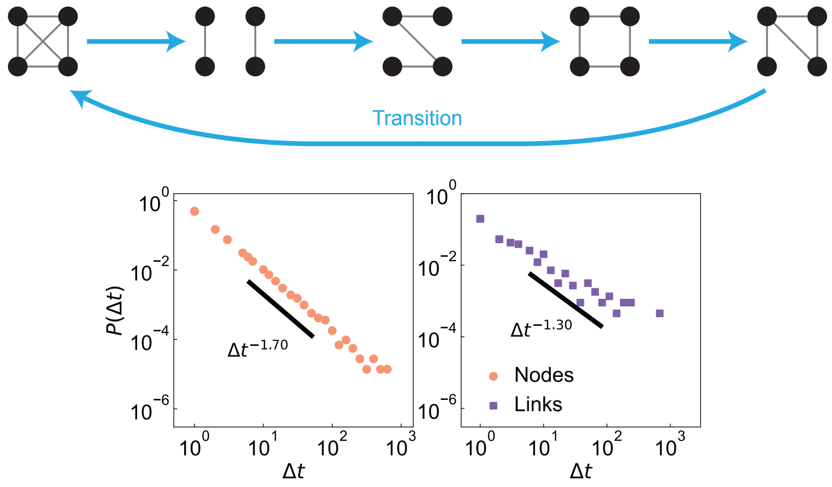

Supplementary Figure 11: Transitions in the underlying topology for four nodes. A completely connected clique of size 4 can periodically transition into distinct cliques of size 2. Depending upon the underlying topology at a given time point, all five edges must be active when all nodes are active (network 1), or two or three, or four edges can be active when all nodes are active (networks 2-5). Our construction successfully generates the targeted burstiness in this time-varying underlying topology.

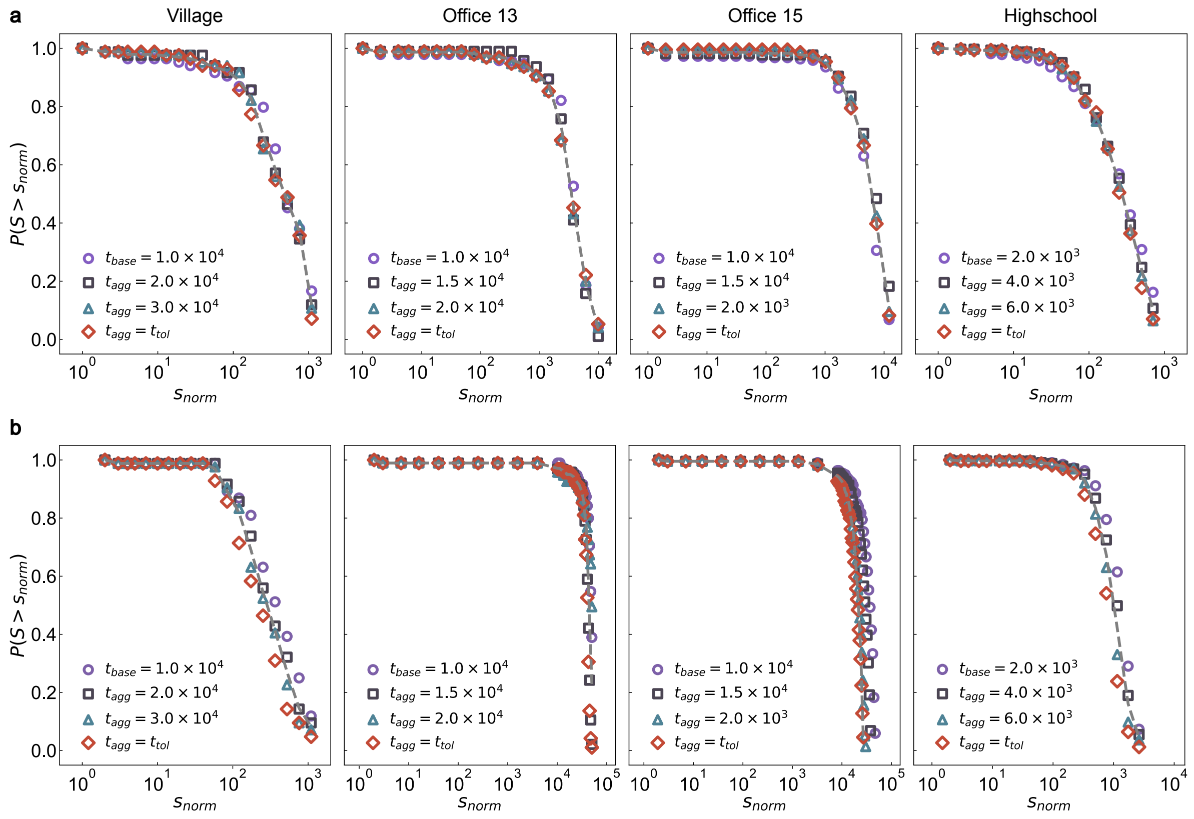

Supplementary Figure 12: Stationary property of node strength distributions in empirical temporal networks. We calculate the survival function of the node strength for the four empirical temporal networks (**a**) and the corresponding algorithmic temporal networks (**b**) in Fig. 3. The normalized distribution of node strength for an aggregation time $t_{agg} > t_{base}$ collapses onto the node strength distribution of the baseline network with the aggregation time t_{base}. The grey dashed lines show the average of the distributions.

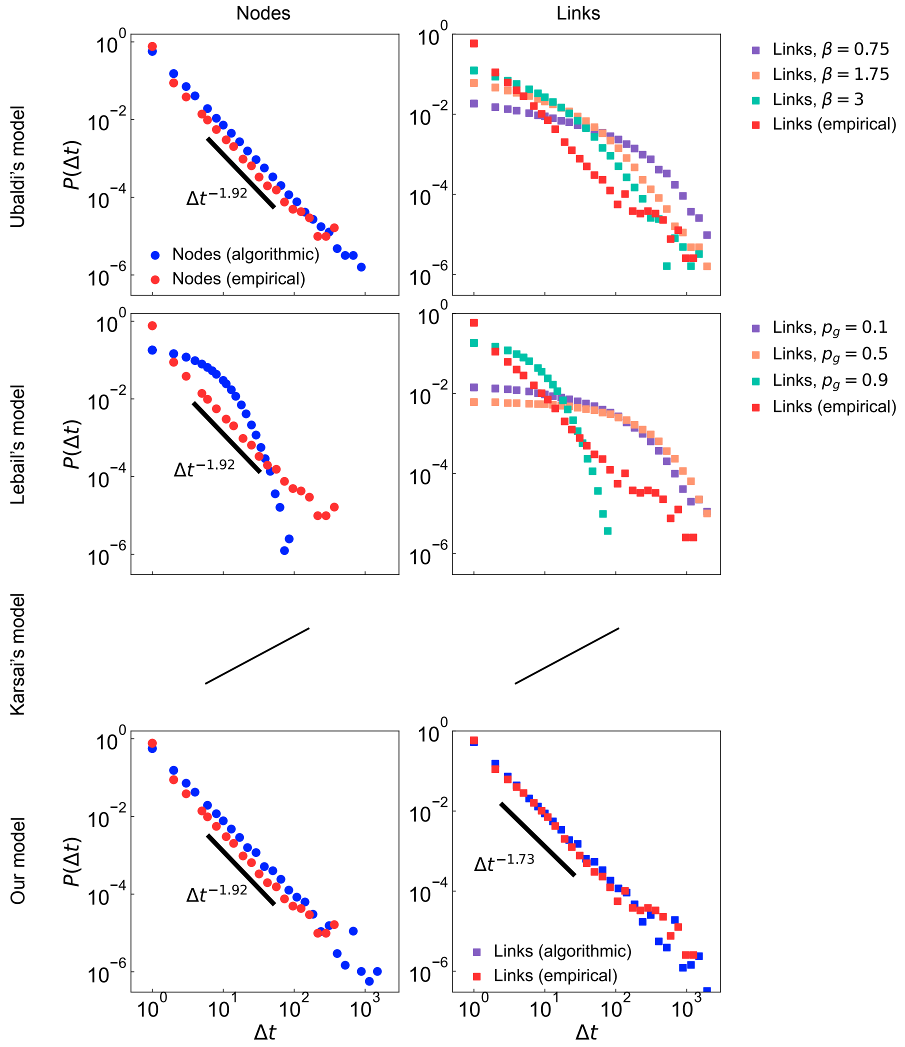

Supplementary Figure 13: The performance of reproducing the simultaneous burstiness of inter-event times for nodes and edges in empirical datasets, using different models. We use the second empirical dataset (Office 13) to test the performance of our model, compared to three previous methods. Ubaldi's model [10] (the first row) successfully reproduces the burstiness of nodes, but it fails to reproduce the burstiness of edges. Lebail's model [11] fails to reproduce the burstiness of both nodes and edges. Karsai's model [12] is applied to single-agent cases, so there is no activity of links whatsoever. Our model reproduces the same level of burstiness as the empirical dataset, in both nodes and edges.

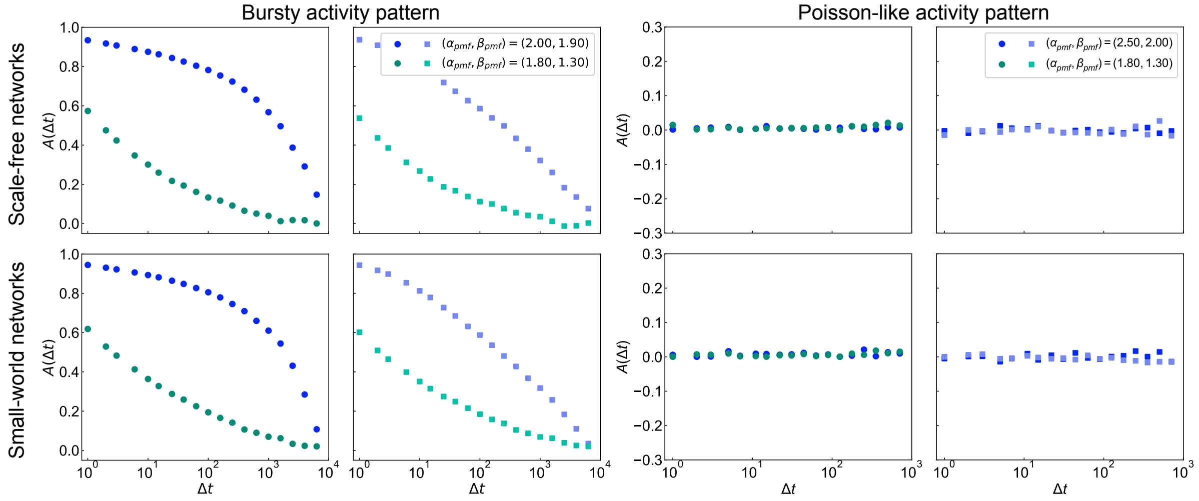

Supplementary Figure 14: Temporal correlation of temporal networks. The relationship between the autocorrelation function, $A(\Delta t)$, and the time interval, Δt, are plotted based on bursty and Poisson-like activity patterns. $A(\Delta t)$ is always positive for any Δt in bursty activity patterns, while $A(\Delta t)$ is approximately 0 in Poisson-like activity patterns.

Supplementary References

[1] Tao, T. *An introduction to measure theory* (American Mathematical Society Providence, 2011).

[2] Rolski, T., Schmidli, H., Schmidt, V., Teugels, J. L. *Stochastic processes for insurance and finance* (John Wiley & Sons, 2009).

[3] Ross, S. M., Kelly, J. J., Sullivan, R. J. et al. *Stochastic processes* (Wiley New York, 1996).

[4] Kolmogorov, A. Sulla determinazione empirica di una lgge di distribuzione. *Inst. Ital. Attuari, Giorn.* **4**, 83–91 (1933).

[5] Myers, J. L., Well, A. D., Lorch, R. F. *Research design and statistical analysis* (Routledge, 2013).

[6] Pearson, K. X. On the criterion that a given system of deviations from the probable in the case of a correlated system of variables is such that it can be reasonably supposed to have arisen from random sampling. *The London, Edinburgh, and Dublin Philosophical Magazine and Journal of Science* **50**, 157–175 (1900).

[7] Barabási, A.-L., Albert, R. Emergence of scaling in random networks. *Science* **286**, 509–512 (1999).

[8] Saavedra, S., Reed-Tsochas, F., Uzzi, B. Asymmetric disassembly and robustness in declining networks. *Proceedings of the National Academy of Sciences of the United States of America* **105**, 16466–16471 (2008).

[9] Goh, K.-I., Barabási, A.-L. Burstiness and memory in complex systems. *Europhysics Letters* **81**, 48002 (2008).

[10] Ubaldi, E., Vezzani, A., Karsai, M., Perra, N., Burioni, R. Burstiness and tie activation strategies in time-varying social networks. *Scientific Reports* **7**, 46225 (2017).

[11] Le Bail, D., Génois, M., Barrat, A. Modeling framework unifying contact and social networks. *Physical Review E* **107**, 024301 (2023).

[12] Karsai, M., Kaski, K., Barabási, A.-L.& Kertész, J. Universal features of correlated bursty behaviour. *Scientific Reports* **2**, 1–7 (2012).
